# Integrative pan-cancer analysis reveals a common architecture of dysregulated transcriptional networks characterized by loss of enhancer methylation

**DOI:** 10.1371/journal.pcbi.1012565

**Published:** 2024-11-18

**Authors:** Jørgen Ankill, Zhi Zhao, Xavier Tekpli, Elin H. Kure, Vessela N. Kristensen, Anthony Mathelier, Thomas Fleischer

**Affiliations:** 1 Department of Cancer Genetics, Institute of Cancer Research, Oslo University Hospital, Oslo, Norway; 2 Institute of Clinical Medicine, Faculty of Medicine, University of Oslo, Oslo, Norway; 3 Oslo Centre for Biostatistics and Epidemiology (OCBE), Faculty of Medicine, University of Oslo, Oslo, Norway; 4 Department of Medical Genetics, Institute of Clinical Medicine, University of Oslo and Oslo University Hospital, Oslo, Norway; 5 Centre for Molecular Medicine Norway (NCMM), Nordic EMBL Partnership, University of Norway, Oslo, Norway; 6 Department of Pharmacy, University of Oslo, Oslo, Norway; Burnet Institute, AUSTRALIA

## Abstract

Aberrant DNA methylation contributes to gene expression deregulation in cancer. However, these alterations’ precise regulatory role and clinical implications are still not fully understood. In this study, we performed expression-methylation Quantitative Trait Loci (emQTL) analysis to identify deregulated cancer-driving transcriptional networks linked to CpG demethylation pan-cancer. By analyzing 33 cancer types from The Cancer Genome Atlas, we identified and confirmed significant correlations between CpG methylation and gene expression (emQTL) in *cis* and *trans*, both across and within cancer types. Bipartite network analysis of the emQTL revealed groups of CpGs and genes related to important biological processes involved in carcinogenesis including proliferation, metabolism and hormone-signaling. These bipartite communities were characterized by loss of enhancer methylation in specific transcription factor binding regions (TFBRs) and the CpGs were topologically linked to upregulated genes through chromatin loops. Penalized Cox regression analysis showed a significant prognostic impact of the pan-cancer emQTL in many cancer types. Taken together, our integrative pan-cancer analysis reveals a common architecture where hallmark cancer-driving functions are affected by the loss of enhancer methylation and may be epigenetically regulated.

## Author summary

In this study, we assessed how changes in DNA methylation–an epigenetic modification—contribute to gene expression alterations across cancer types. Analyzing data from 33 different cancer types, we found that loss of methylation at specific DNA regions, known as CpG sites, is associated with the disruption of key biological processes driving cancer progression, such a cell division, hormone signaling and metabolism. The alterations in DNA methylation were most prominently occurring in enhancer regions, which are gene regulatory regions that can alter gene expression from a distance. Our analysis revealed that these methylation alterations are linked to patient outcomes, suggesting their potential as valuable prognostic markers. Moreover, we identified a recurring pattern of gene dysregulation across different cancer types, demonstrating a common underlying mechanism where the loss of enhancer methylation plays a pivotal role in cancer progression. This study enhances our understanding of how alterations in DNA methylation influence cancer behavior and highlights the possibilities for therapeutic strategies targeting these alterations.

## Introduction

Alterations in transcriptional programs are determinants of cancer cell phenotypes. Such changes occur during carcinogenesis, in which normal cells transform into cancer cells by accumulating genetic and epigenetic alterations. When such alterations occur in regulatory regions of the genome, they alter transcriptional activity and RNA levels, adversely affecting cellular function and initiating carcinogenesis. Epigenetic alterations such as DNA methylation are frequently observed in cancer and are considered a hallmark of many cancer types [1]. Aberrant DNA methylation patterns are commonly observed early during cancer development and have been linked to clinically relevant features such as tumor stage, prognosis and response to therapies [1–3].

During cancer progression, oncogenes tend to be activated through promoter hypomethylation while tumor suppressor genes become inactivated through promoter hypermethylation [4–6]. However, many alterations in DNA methylation occur in more distal intergenic regions. Enhancers are *cis*-acting regulatory regions able to induce gene transcription up to several megabases away from their target gene. To control transcription, enhancers are bound by key proteins such as transcription factors (TFs) and brought into close proximity with their target genes through the formation of chromatin interaction loops [7,8]. TFs are DNA-binding proteins interacting with specific genomic motifs to regulate gene expression. DNA-binding elements tend to be located in unmethylated *cis*-regulatory regions [9]. While some TFs are expressed by most cells, others are cell-type specific and are critical for cell fate determination [10]. Enhancer hypermethylation is associated with transcriptional inactivity, while enhancer hypomethylation is associated with TF binding and transcriptional activation of distal-looped genes [11,12]; however, the role of DNA methylation alterations in enhancer regions during carcinogenesis, as well as the specific transcription factors involved is still poorly characterized.

Hypermethylation and loss of TF binding can alter chromatin interactions in cancer [13]. Moreover, DNA methylation can impair TF binding, and conversely, specific TFs can promote DNA hypomethylation [14]. For instance, TF binding can hinder DNA methyltransferase (DNMTs) access to DNA. Moreover, pioneer TFs, which can engage with heterochromatin to trigger chromatin opening, can interact with histone-modifying proteins and demethylation-competent Ten Eleven Translocation (TETs) enzymes to trigger local DNA demethylation, as demonstrated for FOXA1 and CEBPα [14]. Pioneer factors can also disrupt the activity of DNMTs responsible for maintaining DNA methylation, consequently leading to the loss of DNA methylation during DNA replication and cell division through passive demethylation [14]. Additionally, passive demethylation can occur non-enzymatically when DNA maintenance machinery fails to efficiently restore the lost methylation pattern after each round of cell division. Furthermore, abnormal activity of epigenomic-modifying enzymes can influence the epigenetic landscape and promote intratumoral heterogeneity [15–17]. Our current understanding of the regulatory role of epigenetics on cancer driving functions is still limited, and it is crucial to better characterize the crosstalk between the epigenome and the transcriptome to comprehend tumor phenotypes and their implications for clinical outcomes. Moreover, identifying commonalities and differences in epigenetically dysregulated transcriptional networks across cancer types will pave the way for clinical strategies that may benefit patients with different types of cancer.

We previously performed the expression-methylation quantitative trait loci (emQTL) analysis (termed in Fleischer, Tekpli at al. [18]). In this approach, all available CpGs are tested for correlation to all genes, and all significant correlations are clustered to reveal the importance of enhancer methylation on estrogen signaling and ER-independent proliferation in breast cancer [18,19] and hormone signaling and lipid metabolism in lung cancer [20]. These studies highlighted that epigenetic features of enhancer regions could drive the regulation of gene expression, the activity of TFs at these regions, and chromatin loops. Additionally, we identified emQTL links that derive from the influence of non-cancer cell infiltration on bulk tumor measurements of DNA methylation and gene expression; the identified associations reflected varying immune cell and fibroblast infiltration. In this work, we performed genome-wide emQTL analysis pan-cancer to identify disease-driving epigenetically dysregulated transcriptional networks linked to loss of DNA methylation to better understand how DNA methylation associates with gene expression on a genome-wide scale in multiple cancers and its clinical implications. By integrating DNA methylation and gene expression data from the 33 different cancer types in TCGA, we identified a cell cycle-related community of emQTL characterized by the loss of enhancer methylation situated in transcription factor binding regions for FOSL1/2 and JUN (parts of the AP-1 complex) with concomitant upregulation of proliferation-related genes. The proliferation-related CpGs and genes were functionally connected through predicted chromatin loops. Interestingly, loss of enhancer methylation and upregulation of proliferation-related genes were observed in most cancer types, suggesting that proliferation is epigenetically regulated in several cancer types. Two novel cancer-related communities of emQTL were discovered and linked to the regulation of metabolism and hormone signaling in cancer. Our findings suggest that alterations in enhancer methylation play a key role in regulating hallmark features of cancer. Moreover, we identify specific TFs linked to the regulation of each of these hallmark features. This study provides insights into the regulatory mechanisms of the cancer epigenome on the transcriptome and identifies potential targets for clinical intervention.

## Material and methods

### Patient material and data processing

The Cancer Genome Atlas Program (TCGA) has previously been described [21]. Level 3 DNA methylation (beta values), ATAC-seq (log2((count+5)PM)-qn), and gene expression (log2(fpkm-uq+1)) data from the TCGA-PANCAN dataset were downloaded from the UCSC Xena browser [22]. Methylation probes with more than 50% missing values were removed and the remaining missing values were imputed using the R package impute v1.70.0 (function *impute*.*knn*) for each cancer type independently. For each cancer type, the number of neighbors used in the imputation (k) was set to 10 except for ovarian cancer in which k was set to 3 due to few samples with DNA methylation data from the Illumina HumanMethylation 450k array available.

DNA methylation and gene expression data obtained from primary pancreatic cancer tumors and normal tissue used for validation of the findings in the cell cycle- and hormone-like community were obtained from Nones et al. (GEO accession number GSE49149; Illumine Human Methylation 450k) [23] and from Sandhu et al. (SurePrint G3 Human GE 8x60k microarrays) [24] respectively.

### Cell line data

Raw intensity (IDAT) files with methylation data from the HepG2 and PANC-1 cell lines were retrieved from encode with accession codes GSE128685 and GSE40699 respectively. Beta-values were imputed using the *minfi* R package v1.42.0 (function *getBeta*). DNA methylation data with pre-calculated beta values were obtained from: T-cells (GSE79144), monocytes (GSE68456), leukocytes (GSE69270), B-cells (GSE68456), MDAMB231 (GSE94943) and MCF7 (GSE69188). DNA methylation data from the NCI-60 cell lines were downloaded from the CellMiner database (https://discover.nci.nih.gov/cellminer/home.do) [25].

### Statistical and bioinformatic analyses

All data analyses were performed using the R software version 4.0.2 [26] unless otherwise specified. A p-value<0.05 was considered statistically significant in this study unless otherwise specified. The interaction plot between the emQTL communities was generated using Cytoscape v3.9.1 in which all 192 links from community 4 and 4808 randomly selected emQTL from communities 1–5 were included. Random links were selected by setting the seed parameter to 42 using the R function *set*.*seed*. Each community was labeled using the *AutoAnnotate* v1.4.0 app in Cytoscape. Pan-cancer emQTL analysis R code is available on GitHub (https://github.com/JorgenAnkill/Pan-cancer-expression-methylation-Quantitative-Trait-Loci-analysis). UpSet plots were made using the *UpSet* R package v1.4.0 [27].

### Genome-wide correlation analysis

The TCGA pan-cancer tumor samples with matching DNA methylation and gene expression data available were randomly split into a discovery and validation dataset (R function *sample*, seed = 42). All CpGs with an interquartile range (IQR)>0.1 (191 636 CpGs) and genes with a non-zero IQR (17 265 genes) in the discovery dataset (*n* = 4104) were tested for significant correlations using Pearson’s correlation. Due to the large number of associations, we considered significant only correlations with a Pearson correlation coefficient smaller than -0.5 (corresponding to p-value<1.40e-258), which is much stricter than Bonferroni-corrected p-value less than 0.05 (corresponding to p-value<1.51e-11). Significant CpG-gene associations from the discovery dataset were reanalyzed in the validation dataset (*n* = 4089). Associations were validated if the correlation coefficient was smaller than -0.5.

### Global pan-cancer emQTL network community detection using CONDOR

A clustering algorithm for community detection was applied to the pan-cancer emQTL with negative correlations using the *condor* R package v1.1.1 (function *condor*.*cluster*) [28]. The multilevel community was set as the clustering method and the absolute Pearson correlation coefficient of the pan-cancer emQTL was used as weight. For reproducibility, the initial state of the generator (seed) was set to 42.

### Gene set enrichment analysis

Gene sets used for GSEA were obtained from the Molecular Signatures Database (MSigDB) v.7.4 [29]. Enrichment of genes in each gene set of interest was performed by hypergeometric testing using the C5 gene ontology- and hallmark (H) gene set collections. Enrichment with a BH-corrected p-value<0.05 was considered statistically significant.

### Pathway enrichment analysis

Pathway enrichment analysis was performed on the gene sets obtained from each of the emQTL communities using the Reactome webtool (https://reactome.org) version 89 [30].

### Hierarchical clustering of DNA methylation and gene expression levels

Clustering of DNA methylation and expression data was performed using the R package *pheatmap* v1.0.12 with Euclidean distance and ward.D2 clustering method unless otherwise specified. Expression values were transformed into z-scores before clustering for visualization purposes.

### Genomic annotation

Genome annotation data of *cis*-Regulatory Elements (cCREs) from The Encyclopedia of DNA Elements (ENCODE) [31] was downloaded from the SCREEN webtool (https://screen.encodeproject.org). The registry encompasses 1,063,878 human regulatory regions from 4834 experimental datasets representing 1,518 different cell types and tissues. Nine different cCREs were included in the dataset; “pELS, CTCF-bound”, “dELS, CTCF-bound”, “PLS, CTCF-bound”, “CTCF-only, CTCF-bound”,”dELS”,”DNase-H3K4me3, CTCF-bound”, “pELS”, “PLS” and “DNase-H3K4me3”. In downstream analyses, some of these annotations were collapsed/converted into one as follows: Proximal CTCF-bound enhancer =“pELS, CTCF bound”, Distal CTCF-bound enhancer =“ dELS, CTCF-bound”, CTCF-bound promoter =“ PLS, CTCF-bound” and “DNase-H3K4me3, CTCF-bound”, Promoter =“ PLS” and “DNase-H3K4me3”, Distal enhancer =“ dELS”, pELS =“ Proximal enhancer” and CTCF =“ CTCF-only, CTCF-bound”.

Cell-type specific genome-wide segmentation data from the pancreatic cancer cell line PANC1 and the liver cancer cell line HepG2 was retrieved from Segway (https://segway.hoffmanlab.org). Segway is a method used to annotate the genome using a dynamic Bayesian network model on ChIP-seq data obtained from cell lines and/or tissues [32]. Enrichment of emQTL-CpGs in Segway-defined regulatory regions was assessed by hypergeometric testing (R function *phyper*) using the IlluminaMethylation450k CpGs as background. Adjusted p-values were obtained by Benjamini-Hochberg correction.

### Transcription factor enrichment analysis

Maps of direct TF-DNA interactions were obtained from the UniBind database (https://unibind2018.uio.no) [33]. The UniBind TFBSs correspond to high-confidence direct TF-DNA interactions determined experimentally through ChIP-seq and computationally through position weight matrices (PWMs) from JASPAR [34]. The predicted TFBSs were derived from 1983 ChIP-seq experiments for 231 TFs across cell types and tissues and were predicted to have high PWM scores and be near ChIP-seq peaks.

Since the genomic coordinates of the UniBind database are based on the hg38 reference genome, all CpG positions from the Illumina 450k array were lifted over from hg19 to hg38 using the LiftOver web tool from the UCSC genome browser (https://genome.ucsc.edu) and were extended ±150bp from TFBSs. The CpG positions were then intersected with the TBFR using BEDTools v2.27.1 [35]. Since UniBind maps of TF binding sites are often derived from several ChIP-seq experiments for each TF, we merged the TF binding sites for all ChIP-seq experiments for each TF. Enrichment of CpGs in TFBRs was imputed using hypergeometric testing (R function *phyper*) using the IlluminaMethylation450k CpGs as background. Multiple testing was accounted for using Benjamini-Hochberg correction (R function *p*.*adjust*).

### Cell type enrichment analysis by xCell

Deconvolution of the cellular composition of the tumor samples in the TCGA-PANCAN dataset was performed using the *xCell* [36] r-package version 1.1.0 (function *xCellAnalysis*). xCell is a machine learning algorithm trained on 64 stromal and immune datasets to identify specific cell types from bulk tissue using 10 808 genes as signatures. To assess the link between the xCell-derived cell type scores, the tumor samples were divided into quartile groups based on the xCell score; i.e., the level of infiltration. Differences in DNA methylation at CpGs or expression of genes between the groups were then assessed using the Kruskal-Wallis test.

### Chromatin interactions

Predicted cis-regulatory elements-gene associations were obtained from the GeneHancer database v5.0 [37]. Enhancer to gene associations was obtained from GeneCards Suite Version 5.0 [37]. The genomic coordinates were lifted over from hg38 to hg19 using the LiftOver web tool from the UCSC genome browser (https://genome.ucsc.edu). Predicted Integrated Methods for Predicting Enhancer Targets (IM-PET) interactions from the PANC-1, HepG2, A549, HCC1954, MCF7, HCT116, HELA, and K562 cell lines were retrieved from the 4D Genome data portal (https://4dgenome.research.chop.edu) [38]. IM-PET is a computational algorithm used to predict chromatin interaction loops between enhancers and promoters by using genomic features including distance restrictions, enhancer-target promoter activity profile correlation, TF and target promoter correlation, and co-evolution of enhancer and target promoter [39]. Experimentally defined Chromatin Interaction Analysis by Paired-End sequencing (ChIA-PET) data defining long-range chromatin interactions in the MCF7 breast cancer cell line was obtained from ENCODE (Accession number ENCR000CAA [40]). BEDTools v2.27.1 was used to intersect gene and CpG positions with the genomic intervals defining the feet of the chromatin loops for the GeneHancer, IM-PET and ChIA-PET data. An emQTL was considered in a chromatin loop if the CpG and gene were found in different feet of the same chromatin loop. To avoid bias from the array design, enrichment of emQTL in chromatin loops was calculated using a hypergeometric test (R function *phyper*) using all possible loops between CpGs and genes (i.e., CpG-gene pairs) on the same chromosome on the 450K array as background. Visualization of the chromatin interactions was made using the *Gviz* v1.40.1 [41] and *GenomicRanges* v1.48.0 [42] R packages and genomic interactions were visualized in Gviz using the *GenomicInteractions* v1.30.0 [43] R package.

### Tumor purity data

Allele-Specific Copy Number Analysis of Tumors (ASCAT) [44] is a computational tool that estimates tumor purity by inferring the fraction of tumor cells in a sample. This is done by analyzing the proportion of tumor-specific and normal-specific allele frequencies in DNA copy number data. ASCAT compares the observed DNA copy number data to a reference profile, taking into account DNA contamination, ploidy of cancer cells and the presence of subclonal populations of cells with different copy number alterations. Tumor purity estimates for the TCGA samples were obtained from the NCI Genomic Data Commons (GDC) data portal upon request (https://portal.gdc.cancer.gov) [45].

### Assessing the predictive power of the pan-cancer emQTL

The ability of the pan-cancer emQTL to predict prognosis was assessed by building Cox regression with ridge penalty to contract models for survival predictions by estimating the hazard function (Ridge Cox model) [46]. Principal component analysis (PCA) was performed on the z-scores of the expression and methylation data for each cancer type in TCGA using the R package *stats* v3.6.2. In each community, the first two principal components (PCs) of emQTL-CpGs and the first two PCs of the emQTL-genes were extracted, thus 24 PCs were extracted from the six communities. We regressed patients’ survival in each cancer type on the 24 PCs using the Ridge Cox model (R package *glmnet* v4.1.6). The survival prediction metric Uno’s C-index (R package survAUC v1.1.1) was obtained using 50 5-fold (80–20 split) cross-validation to overcome overfitting. The seed parameter was initialized as 123 using the R function *set*.*seed*. Survival plots were made using the *survminer* R package v0.4.8.

To generate Kaplan-Meier survival curves, we repeated the analysis using a 60–40 split between training and test data, and the trained model was applied to the test data. Kaplan-Meier survival curves and Log-rank tests were performed using the R functions *Surv* and *survfit* from the R package *survival* v3.5.3.

## Results

### Identification of pan-cancer expression-methylation quantitative trait loci (emQTL)

To identify robust associations between DNA methylation and gene expression in *cis* and *trans*, we correlated genome-wide levels of DNA methylation and gene expression from the TCGA-PANCAN dataset (see Material and Methods; S1 Fig for workflow outline). The TCGA pan-cancer dataset was split into a discovery (n = 4104) and a validation cohort (n = 4089; see Material and methods). 1 192 567 (87%) out of the 1 363 565 significant CpG-gene associations discovered were significant in the validation cohort. Most correlations had negative Pearson coefficient values (64.8% negative vs 35.2% positive; S2 Fig), indicating that methylation at a CpG increases when the level of expression for the correlated gene decreases, which can reveal a potentially putative inhibitory effect of CpG methylation on gene expression. The validated emQTL included 5472 genes and 45 406 CpGs. We found *cis*-associations (same chromosome; 83 035 associations) to be significantly enriched compared to *trans* associations (1 037 323 associations; hypergeometric test p-value<2.2e-16; fold enrichment (FE = 1.42). The validated associations are hereafter called emQTL. Although causality on gene expression regulation cannot be determined from statistical correlations, the emQTL provides insight into the overall degree of coordination between CpG methylation and gene expression globally, considering both potential direct and indirect regulation.

### Characterization of the pan-cancer emQTL by bipartite network analysis reveals communities with distinct biology

**Fig 1:**
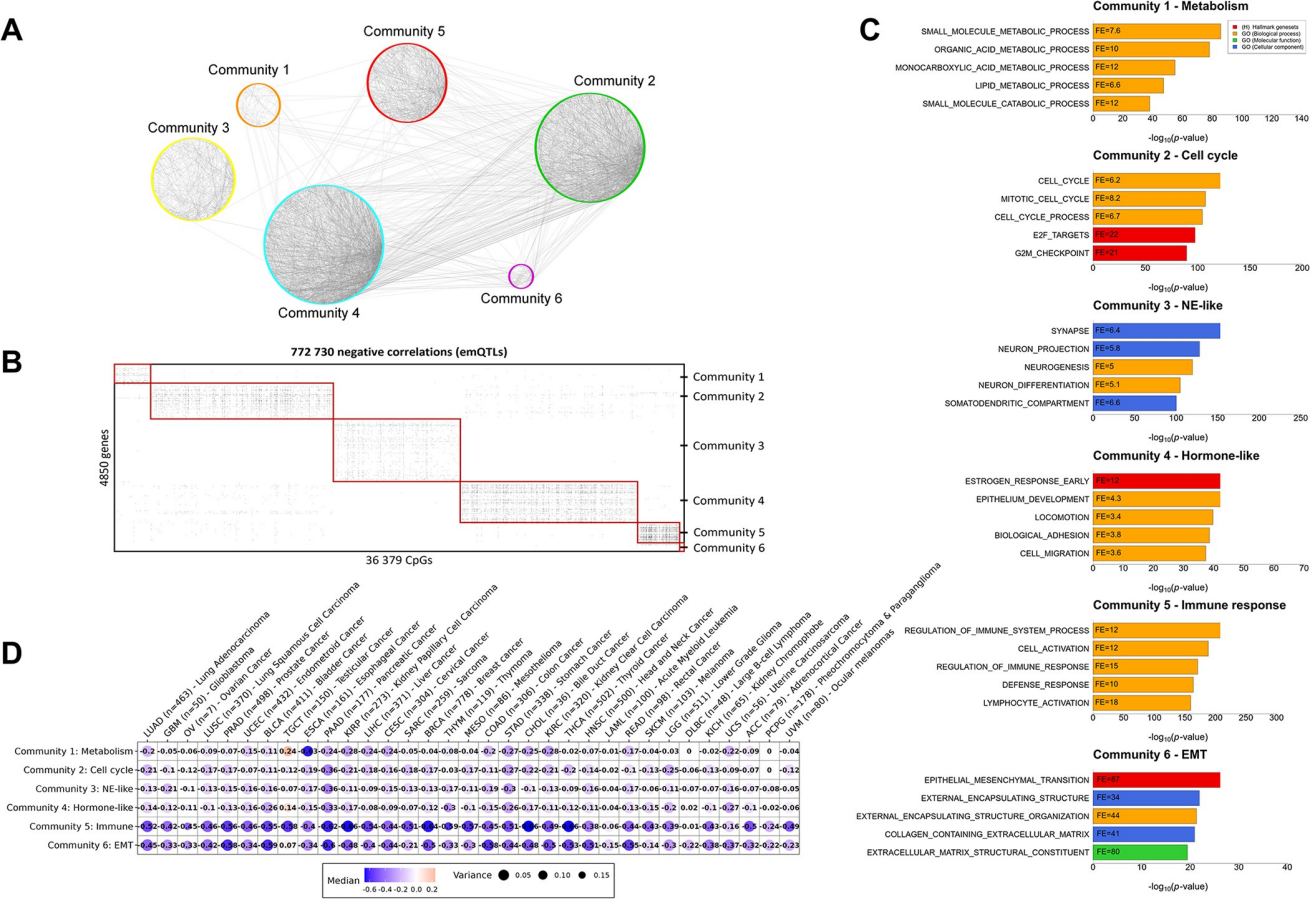
Characterization of the pan-cancer emQTL. (A) Interaction plot showing the emQTL associations using Cytoscape v.3.9.1 [47]. For clarity of visualization, only 5000 intra- and inter-community associations are shown (4808 randomly selected emQTL related to communities 1 to 5 and all 192 emQTL related to community 6). (B) Heatmap showing the pan-cancer emQTL found in the TCGA-PANCAN dataset (772 730 emQTL) sorted by emQTL community in which black points indicate significant CpG-gene associations (emQTL). Rows represent genes and the columns represent CpGs involved in emQTL. (C) GSEA of the genes in communities 1 to 6 using the Hallmark and gene ontology C5 (GO biological process, GO molecular function, and GO cellular component) gene set collections from the MSigDB [48]. The bar length represents the -log10-transformed Benjamini-Hochberg (BH) corrected p-value obtained by hypergeometric testing. Only the top 5 most significantly enriched gene sets for each community are shown. (D) Dot plot showing the median Pearson coefficient obtained by reanalyzing the pan-cancer emQTL in each community for the 33 cancer types separately. The color of the dot represents the sign of the median correlation coefficients (i.e., median negative correlation coefficient in blue and median positive correlation coefficient in red). Dot size denotes the variance of the emQTL correlation coefficients.

To identify coordinated emQTL, we performed bipartite network analysis using Complex Network Descriptions Of Regulators (CONDOR) [28], identifying communities of highly connected emQTL-CpGs and emQTL-genes. Since our study focused on the events where DNA methylation had a putative inhibitory effect on gene expression, we considered negative correlations in the community detection analysis (772 730 emQTL). We detected six pan-cancer emQTL communities (Fig 1A and 1B and S1A and S1B Table). To elucidate the biological functions of the emQTL communities, we performed gene set enrichment analyses (GSEA) using gene sets obtained from the Molecular Signatures Database (MSigDB) [29]. We found the communities to be enriched for genes involved in metabolic processes (Community 1), cell cycle (Community 2), neuroendocrine-like (NE-like) functions (Community 3), hormone-signaling (Community 4), immune response (Community 5) and epithelial-mesenchymal transition (EMT; Community 6; Fig 1C and S1C Table). Pathway enrichment analysis was performed and validated that the genes in each emQTL community were enriched in their respective functions as identified in the GSEA. However, significant enrichment was not observed for the hormone-signaling community, which is likely due to the lack of specific pathways for hormone-signaling in Reactome (S1D Table and S3 Fig). The number of CpGs covering the genes in the metabolism, cell cycle and hormone like communities showed similar distribution as all genes in the genome, showing that our analysis does not introduce notable bias potentially caused by CpGs being unevenly distributed across genes (S4 Fig).

We re-analyzed all emQTL within each cancer type and showed that there is a correlation within most cancer types for most communities (i.e., high methylation is associated with low expression also within individual cancer types), which points to that the identified biological functions are linked to epigenetic dysregulation both within and across cancer types (Fig 1D).

### The emQTL-CpGs reside in *cis* regulatory regions

**Fig 2:**
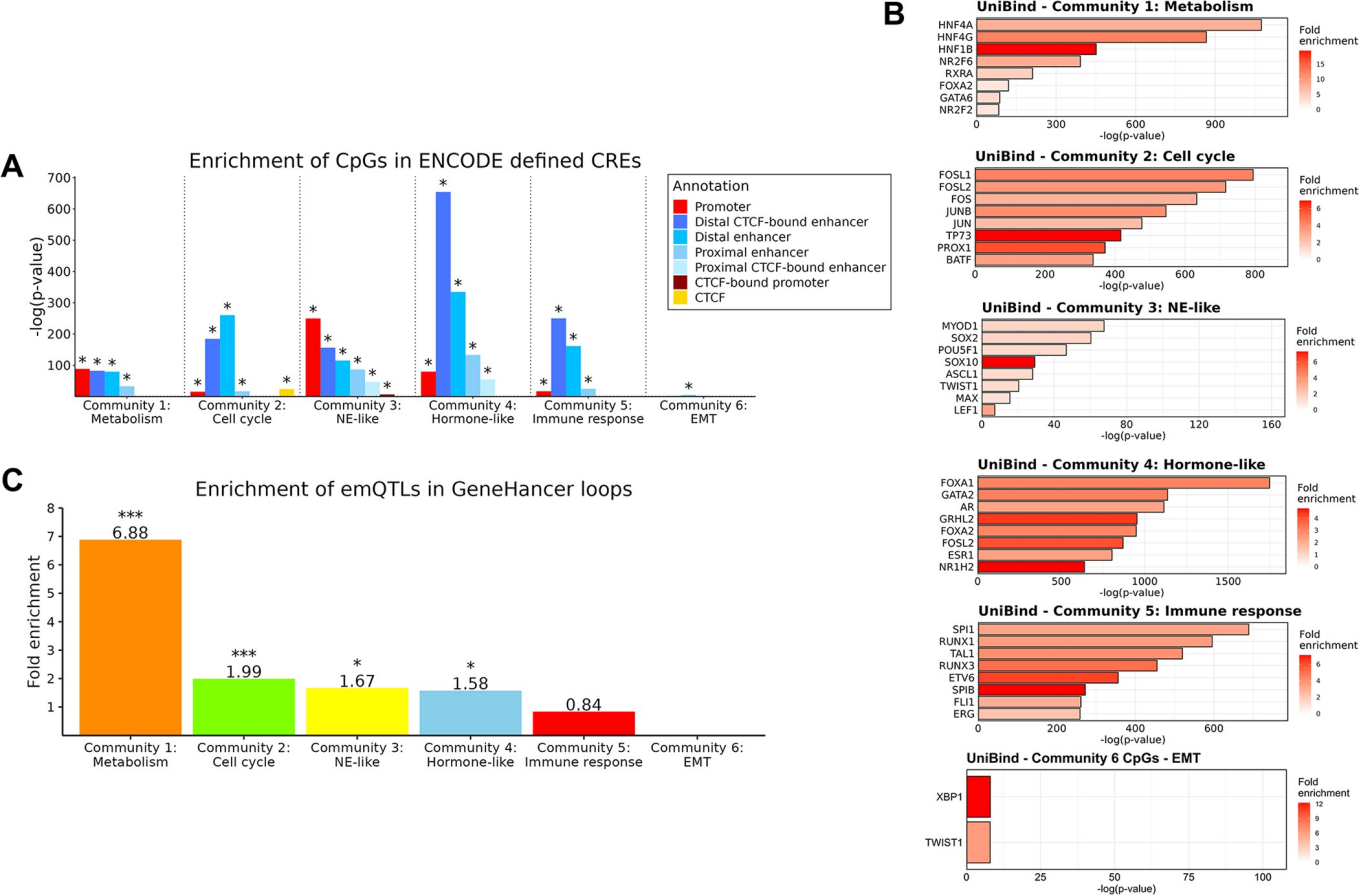
Genomic location of the pan-cancer emQTL. (A) Enrichment of emQTL-CpGs in ENCODE-defined cCREs. The height of the bars represents the log-transformed BH-corrected p-value obtained by hypergeometric testing using the 450k probes as background. Significant enrichments (BH-corrected p-value<0.05) are marked with an asterisk. (B) Enrichment of emQTL-CpGs in TFBR by emQTL community according to UniBind [33]. The length of the bars corresponds to the log-transformed p-values obtained by hypergeometric testing. Only the top most significantly enriched TFs are shown in each plot. (C) Bar plot showing the enrichment of emQTL from each emQTL community in GeneHancer loops. The height of the bars represents the fold enrichment measured as the ratio between the frequency of emQTL found in the head and tail of GeneHancer loops over the expected frequency if such overlaps were to occur at random. Statistically significant enrichments BH-correction obtained by hypergeometric testing are marked with an asterisk (* is p<0.05, ** is p<0.001, *** is p<0.0001). See **Materials and methods** for a detailed description of the calculation of enrichment.

To assess the functional context of the pan-cancer emQTL-CpGs, we determined their enrichment in specific categories of regulatory regions by considering the pan-tissue candidate *cis*-Regulatory Elements (cCREs) predicted by ENCODE [31]. We found the emQTL-CpGs from all communities significantly enriched in enhancer regions (Fig 2A and S1E Table). Moreover, the CpGs in communities 1 to 5 were enriched in promoter regions. Since TFs can bind to promoter and enhancer regions to modulate gene expression, we aimed to identify which TFs may bind to these regions using TF-DNA interaction data obtained from the UniBind database [33]. We found the emQTL-CpGs in each community to be enriched in TF binding regions (TFBR) of TFs associated with functions coherent with the GSEA of the communities (Fig 2B and S1F Table). In the metabolism community, CpGs were enriched in binding regions of hepatocyte nuclear factors (HNFs) and NR2F2/6 known to be associated with metabolism. In the cell cycle community, CpGs were enriched in binding regions of Fos and Jun family TFs known to be involved in proliferation. In the hormone-like community, CpGs were enriched in binding regions of ER and AR, as well as FOX and GATA family TFs, known to be important in hormone signaling in cancer. Taken together, our analysis highlight the cancer-relevant role of proliferation-, metabolism- and hormone signaling-associated TFs. We also observed that the enriched TFs in each community shared binding regions, especially in the cell cycle bicluster (S5 Fig).

Enhancers can regulate gene expression through the binding of TFs and the formation of chromatin loop interactions with their target gene. As all emQTL communities were enriched for enhancer regions, we assessed whether the emQTL-CpGs and genes were linked through predicted loops from GeneHancer [37]. Our analysis revealed that the emQTL in communities 1 to 4 were significantly enriched in GeneHancer loops (Fig 2C, Hypergeometric test p-value = 5.50e-11, 2.74e-05, 3.59e-03, 7.97e-04 respectively; S6 Fig and S1G Table). No statistically significant enrichment in loops was observed for the emQTL in the immune and EMT communities. To further validate the enrichment in chromatin loops, we utilized IM-PET data from eight cancer cell lines and assessed enrichment for CpG-gene pairs in all communities (S6 Fig; S1G Table). These data show that CpG-gene pairs in the cell cycle-, metabolism- and hormone-like communities were enriched in chromatin loops in most of the available cell lines. Finally, in experimentally-defined chromatin interaction data (ChIA-PET) from the MCF7 breast cancer cell line, we observed enrichment in loops for all communities except the EMT community (S6 Fig and S1G Table). The results from the emQTL communities are summarized in Table 1.

**Table 1:** Summary of the pan-cancer emQTL communities.

Community	Number of CpGs	Number of genes	GSEA function (MSigDB)	Enriched ENCODE-defined cCRE	Enriched UniBind TFs	Enrichment in GeneHancer associations
**1**	2207	447	Metabolism	Promoters, enhancers	HNF4A/G/B and RXRA [49–52]	Enriched
**2**	11 517	892	Cell cycle	Promoters, enhancers, CTCF binding sites	FOSL1/2 and JUN [53–55]	Enriched
**3**	8136	1560	NE-like	Promoters, enhancers	MYOD1, SOX2, POU5F1 [56–58]	Enriched
**4**	11 166	1067	Hormone-like	Promoters, enhancers	ESR1 [59] and AR [60]	Enriched
**5**	2674	529	Immune response	Promoters, enhancers	SPI1 [61], RUNX1/3 [62, 63] and TAL1 [64]	Not significant
**6**	31	43	EMT	Enhancers	TWIST1 and XBP1 [65, 66]	Not significant

### The pan-cancer emQTL can predict survival in many cancer types

**Fig 3:**
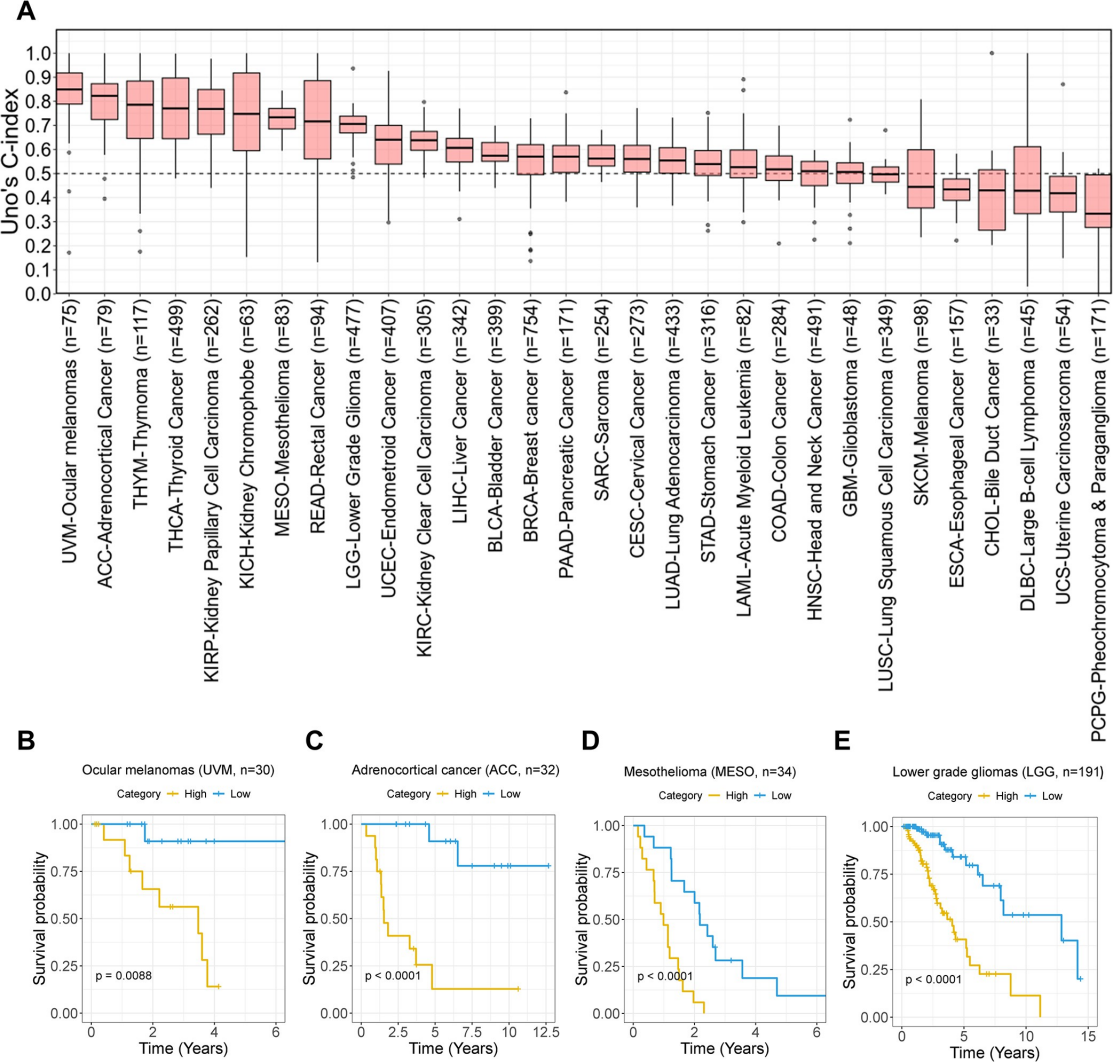
Survival prediction of the pan-cancer emQTL. (A) The pan-cancer patients were randomly split into 80% training and 20% test data 50 times. Each box shows Uno’s C-indexes estimated from the randomly split 20% test data. Due to few samples for ovarian cancer (n = 8) and high censoring in prostate- and testicular cancer, these cancer types were not included in the analysis. Kaplan-Meier survival curves and log-rank tests show the ability of the pan-cancer emQTL to discriminate patients into high- and low-risk groups in regards to survival in ocular melanoma (B), adrenocortical cancer (C), mesothelioma (D) and lower grade glioma (E). Patients were divided into two categories based on the risk scores obtained from the Cox models with ridge penalty. The pan-cancer patients for each cancer type were randomly split into 60% training and 40% test data.

To assess the ability of the pan-cancer emQTL to predict prognosis, we built Cox regression with a ridge penalty to construct models for survival predictions by estimating the hazard function (Ridge Cox model) [46]. Specifically, we performed principal component analysis (PCA) on the z-scores obtained from methylation and expression data for the emQTL-CpGs and genes to standardize the data on the same scale (see Material and Methods). In each community, the first two principal components (PCs) of emQTL-CpGs and -genes were extracted. We regressed patients’ survival in each cancer type by Ridge Cox model and obtained Uno’s C-index. For comparison, we extracted the first two PCs from the emQTL-CpGs and emQTL-genes respectively, i.e., without considering emQTL communities, and regressed patients’ survival in each cancer type on the four PCs from all the emQTL communities by Ridge Cox model. Uno’s C index was estimated to evaluate the models in which a value below 0.5 indicates a poor model and a value above 0.7 indicates a good model. The PCs from the pan-cancer emQTL considering the six communities show the predictive power of patients’ survival (Fig 3A), and had significantly higher C-indexes than the four PCs from the pan-cancer emQTL without considering the communities (p-value = 1.41e-11; S7 Fig). Fig 3B–3E shows examples of differences in survival outcomes between the predicted high- and low-risk group for the ocular melanoma, adrenocortical cancer, mesothelioma, and lower-grade glioma (C-index = 0.81, 0.75, 0.75 and 0.68 respectively). Altogether, our analysis highlights the ability of the pan-cancer emQTL to predict survival outcomes. We hypothesize that the emQTL has an important role in cancer-related regulation of transcriptional networks.

### The cell cycle community highlights important processes of cancer pathogenesis

**Fig 4:**
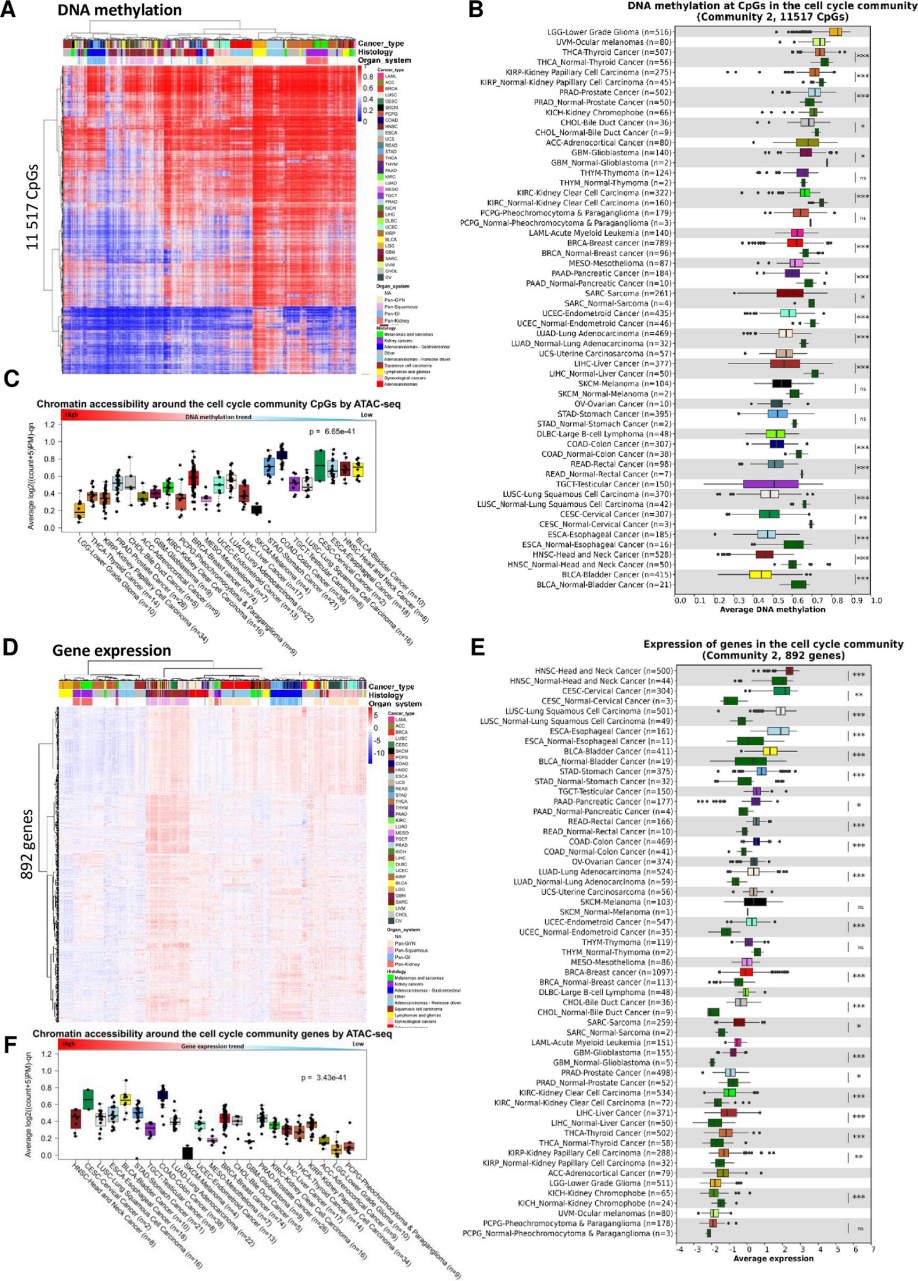
DNA methylation at CpGs and expression of genes in the cell cycle community. (A) Hierarchical clustering of DNA methylation levels at CpGs in the cell cycle community in the TCGA-PANCAN dataset (n = 8229). Rows represent CpGs and columns represent tumor samples. Unmethylated CpGs are shown in blue, and methylated CpGs are in red. Histopathological features including cancer types, organ system and histology are indicated in the columns. (B) DNA methylation at the cell cycle community CpGs by cancer type in the TCGA-PANCAN dataset. Normal samples are included for those cancer types with data available from TCGA. BH-corrected Wilcoxon-rank sum test p-values are denoted. (C) Box plot showing the chromatin accessibility (based on ATAC-seq) in the region at the cell cycle community CpGs obtained from the TCGA-PANCAN dataset. A high value indicates open chromatin, while a low value indicates more compact chromatin. The cancer types are ordered by the median DNA methylation levels at the CpGs in the cell cycle community. (D) Unsupervised hierarchical clustering of gene expression levels of the cell cycle community genes in the TCGA-PANCAN dataset (n = 9875). Genes are found in the rows and the tumor samples are in the columns. Red indicates high expression and blue indicates low expression. Annotations of cancer type and organ system are included. (E) Average expression of the cell cycle community genes by cancer type. As a comparison, expression values from normal adjacent tissues are included if available for the specific cancer type. BH-corrected Wilcoxon rank-sum test p-values are denoted. (F) shows the chromatin accessibility around the cell cycle community genes in the TCGA-PANCAN dataset obtained by ATAC-seq. The cancer types are ordered by the median expression levels of the genes in the cell cycle community for each particular cancer type.

To shed light on the pathogenic molecular processes associated with the cell cycle community, we performed hierarchical clustering of DNA methylation levels at CpGs and expression of genes in the cell cycle community using the TCGA-PANCAN dataset. We found the methylation and expression profiles to reflect the tissue of origin of the tumors (Fig 4A and 4D; Chi-square test p-value<2.2e-16 (three patient subclusters)) which is consistent with previous reports [67]. We also categorized tumors into broader histological/tissue types classes, and observe a clear organization of tumors according to this classification. Moreover, we observed that the cell cycle community CpGs are hypomethylated in most cancer types compared to normal adjacent tissue (Fig 4B). The hypomethylation is specifically observed at the TFBRs of FOSL1/2 and JUN (S8A Fig). We further assessed whether the CpGs and genes associated with the cell cycle community were located in open chromatin regions using ATAC-seq data [68]. As expected, the cancer types harboring open chromatin around the cell cycle community CpGs were also the cancer types with the lowest level of DNA methylation at these sites (Fig 4C). Concomitantly, the genes associated with the cell cycle community show significantly higher expression in tumor cells than in normal cells for most cancer types (Fig 4E). Finally, the cancer types with the highest expression level of the cell cycle community genes correspond to the ones with the highest open chromatin signal (Fig 4F). The top five hub genes (genes with most associations to CpG methylation) were *CEP55*, *TUBA1C*, *FAM83D*, *AURKA* and *CDCA8* (S1B Table), all linked to regulation of the cell cycle and microtubule cytoskeleton organization during mitosis. These lines of evidence show that the cell cycle community CpGs are hypomethylated in most cancer types and are found in accessible enhancer regions forming interactions with transcription start site (TSS) within open chromatin regions regulating cell cycle-related genes.

### The hormone-like and metabolism community links alteration in enhancer methylation to variations in gene expression through looping

Hierarchical clustering of DNA methylation levels at the hormone-like and metabolism communities CpGs separated the tumors based on the tissue of origin, but also indicated variability in DNA methylation within each cancer type (S9A and S9B Fig). The CpGs in both communities were significantly enriched in enhancer regions and were less methylated in most cancer types compared to normal adjacent tissue (S9C and S9D Fig). Loss of methylation was also observed specifically at the TFBRs of FOXA1, ESR1 and AR at the CpGs in the hormone-like community and at the HNF4A/G and RXRA TFBRs in the metabolism community (S8B and S8C Fig). By assessing the chromatin accessibility around the hormone-like and metabolism community CpGs, we found lower DNA methylation levels at the CpGs to be associated with more accessible chromatin (S9E and S9F Fig). Similarly, to the methylation data, the expression of genes in the hormone-like and metabolism-related communities clustered according to the tissue of origin, and variability in expression within the cancer types was also observed (S10A and S10B Fig). We next compared the expression of the hormone-like and metabolism community genes between tumor and normal tissue and found genes in both communities to be significantly higher expressed in tumor tissue compared to normal adjacent tissue for most cancer types (S10C and S10D Fig). High expression of the hormone-related and metabolism-related genes was also associated with more accessible chromatin (S10E and S10F Fig). The top five hub genes for the hormone-like community were *KRT18*, *JUP*, *KRT8*, *SH2D3A* and *PDLIM1* (S1B Table); *KRT18* and *KRT8* are luminal markers in breast cancer while *JUP* (plakoglobin), *SH2D3A* and *PDLIM1* are relatively undescribed in the context of cancer. The top five hub genes for the metabolism community were *HNF4A*, *SLC39A5*, *AMN*, *UGT2A3* and *SLC17A4* (S1B Table). *HNF4A* encodes a TF involved in development of beta cells in the pancreas, and *AMN* (amnionless) is involved in vitamin uptake. The roles of *SLC39A5*, *UGT2A3* and *SLC17A4* are relatively undescribed in cancer. The emQTL in both communities were enriched in inferred GeneHancer enhancer-promoter loops, thereby suggesting a possible functional regulation of hormone-related and metabolism genes. Altogether, our findings suggest that enhancer methylation is linked to the upregulation of hormone- and metabolism-related genes through chromatin looping across cancer types.

Adipocytes are a prominent cell type of the tumor microenvironment in solid tumors and express high levels of metabolic genes involved in energy storage and mobilization. To ensure the signal from the metabolism community was not caused by infiltration of non-tumor cells such as adipocytes, we correlated the average DNA methylation levels at the CpGs genes in the metabolism community with the ASCAT tumor purity estimates [44]. No statistically significant correlation was observed between methylation and tumor purity in TCGA (S11 Fig, p-value = 0.90). A significant correlation was observed between the average expression of the metabolism genes and tumor purity (p-value = 8.54e-06, r-value = -0.05); however, the Pearson correlation coefficient r was close to zero, suggesting that the variations in methylation and expression profiles of the metabolism community are caused by differences between the cancer cells.

### The immune community reflects variations in the tumor microenvironment

Unlike the DNA methylation levels at the CpGs and expression levels at the genes in the cell cycle, metabolism, and hormone-like communities, CpGs and genes in the immune community did not segregate the tumors equally well according to cancer type (S12A–S12D Fig). Since immune cells are prominent in the tumor microenvironment and have different DNA methylation and gene expression profiles compared to cancer cells, we hypothesize that this community of emQTL was influenced by and reflects variation in immune cell infiltration in tumors. To address this, we used the xCell deconvolution [36] algorithm to quantify the level of immune infiltration using gene expression data from TCGA. We divided the tumors into quartile groups based on the level of immune infiltration. We observed a significant difference in DNA methylation levels at the CpGs in the immune community between the quartile groups in which low methylation at CpGs in the immune-community was associated with high immune infiltration and high DNA methylation with low infiltration (S13A Fig, p-value<2.2e-16). Furthermore, DNA methylation levels at the CpGs in the immune community obtained from isolated immune cells, such as leukocytes, monocytes, T-cells, and B-cells, reflected similar methylation levels at the CpGs as the tumors with high immune infiltration. In contrast, cancer cell lines from the National Cancer Institute (NCI-60) Human Tumor Cell Lines Screen showed similar methylation levels as the quartile group with the lowest infiltration. One exception was leukemia cell lines which is reasonable as the cell of origin for this cancer type is lymphocyte and therefore shares similarities with the infiltrating immune cells. We also observed a positive association between immune infiltration and expression of genes in the immune community, i.e., high expression of the immune community genes is linked to high immune infiltration and vice versa (S13B Fig). To further confirm the link between immune infiltration and the immune community, we obtained copy-number-derived tumor purity estimates from ASCAT [44] and assessed the link between the xCell-derived immune score and tumor purity. We found a negative association between tumor purity and immune cell infiltration, i.e., low tumor purity is associated with high infiltration of immune cells, and vice versa (S13C Fig). Altogether, these results suggest that the emQTL in the immune community is caused by variations in immune cell infiltration.

### The EMT community reflects varying fibroblast infiltration in tumors

The smallest emQTL community was the EMT community consisting of 31 CpGs and 43 genes representing 120 emQTL. Similar to the immune community, DNA methylation at the CpGs and expression of genes did not segregate the tumors according to cancer types compared to the cell cycle, metabolism and hormone-like communities (S14A–S14D Fig). Since fibroblasts are a prominent mesenchymal cell type of the tumor microenvironment of many solid tumors and carry out functions such as migration and remodeling of the extracellular matrix (ECM) [69], we assessed whether the EMT community could arise from heterogeneity in tumor composition in regards to fibroblast infiltration. We employed the xCell deconvolution tool [36] to estimate the relative quantity of fibroblast infiltration in each tumor sample using gene expression. By dividing the tumor samples into quartile groups based on the severity of fibroblast infiltration, we found that expression of the EMT community genes was positively associated with fibroblast infiltration (S15A Fig, p<2.2e-16), i.e., high expression of EMT community genes is linked to high fibroblast infiltration. We also observed a significant negative association between DNA methylation and fibroblast infiltration (S15B Fig, p-value<2.2e-16). Furthermore, the DNA methylation levels of the EMT community CpGs in tumors with severe fibroblast infiltration showed similar methylation profiles as purified fibroblasts. Conversely, all tumor cell lines from the NCI-60 panel showed methylation levels similar to the low infiltration group. To further confirm that the EMT community was linked to fibroblast infiltration, we assessed whether the quartile groups based on the severity of fibroblast infiltration were linked to the ASCAT tumor purity estimates. A significant and negative association was observed between fibroblast infiltration and tumor purity, i.e., high fibroblast infiltration is linked to low tumor purity, and vice versa (S15C Fig). Taken together, our findings suggest that the EMT community of emQTL is linked to heterogeneity in the severity of fibroblast infiltration in tumors.

### The NE-like community reflects differences in the tissue of origin and heterogeneity in the tumor microenvironment

To study the differences in terms of methylation and expression of the NE-like community CpGs and genes, we performed unsupervised hierarchical clustering of DNA methylation and gene expression levels of the CpGs and genes in the NE-like community. We found the DNA methylation and expression profiles of the CpGs and genes in the NE-like community to cluster by cancer type (S16A and S16B Fig). Among the cancer types, the neuron-related cancer types such as lower-grade glioma (LGG), pheochromocytoma & paraganglioma (PCPG), and glioblastomas (GBM) had pronouncedly different DNA methylation and gene expression profiles compared to the other cancer types. These cancer types showed the highest expression of the NE-like community genes and the lowest DNA methylation at the CpGs in the NE-like community (S16C and S16D Fig). Moreover, these cancer types had less compact chromatin around the NE-like community CpGs and genes than other cancer types (S16E and S16F Fig).

Altogether, our findings suggest that the NE-like community is associated with the cell of origin. The brain-related cancer types have upregulated neural-related genes through low DNA methylation at enhancers. However, this cannot fully explain this community, as there are also associations between DNA methylation at the CpGs and expression of the genes in the NE-like community within non-brain related cancer types (Fig 1D). The methylation and expression levels also differ between tumor and normal adjacent tissue for most cancer types (S16C and S16D Fig). To assess whether this could be caused by variations in the tumor microenvironment, we obtained the tumor microenvironment score from xCell [36]. We assessed the link between DNA methylation of CpGs and the expression of genes in the NE-like community to the severity of infiltration. Due to the prominent differences in methylation and expression profiles between the neuron-like cancer types (LGG, PCPG, and GBM) and the other cancer types, we assessed the link between methylation, expression, and tumor infiltration in the neuron-like (NL) and non-neuron-like (NNL) tumors independently. We found the expression of the NE-like community genes to be positively associated with the microenvironment score from xCell in the NNL tumors (S17A Fig; p-value = 1.29e-121). Moreover, a negative association between the tumor microenvironment score and DNA methylation was observed for the NNL, and the methylation levels from the NCI-60 cancer cell lines reflected the methylation levels in the low infiltration group (S17B Fig; p-value = 3.58e-110). A negative association was observed between the tumor microenvironment score and tumor purity (S17C Fig). Altogether, our findings suggest that the variability in DNA methylation at the CpGs and expression of genes in the NE-like community within the cancer types are caused by variations in the cell composition of the tumor microenvironment in the NNL cancer types. For the NL tumors, there was a negative association between the tumor microenvironment score and expression of the genes in the NE-like community (S17D Fig, p-value = 3.31e-70) and a positive association with the level of DNA methylation (S17E Fig). A negative association between the tumor microenvironment score and tumor purity was also observed, and the main difference in purity was between the tumors with severe infiltration and those with low, moderate, and high infiltration (S17F Fig). Altogether this suggests that the NE-like community is mainly caused by differences in the tissue of origin between the tumors and that differences in tumor composition cause variability in expression and DNA methylation within the cancer types.

### Identification of cancer-driving emQTL

**Fig 5:**
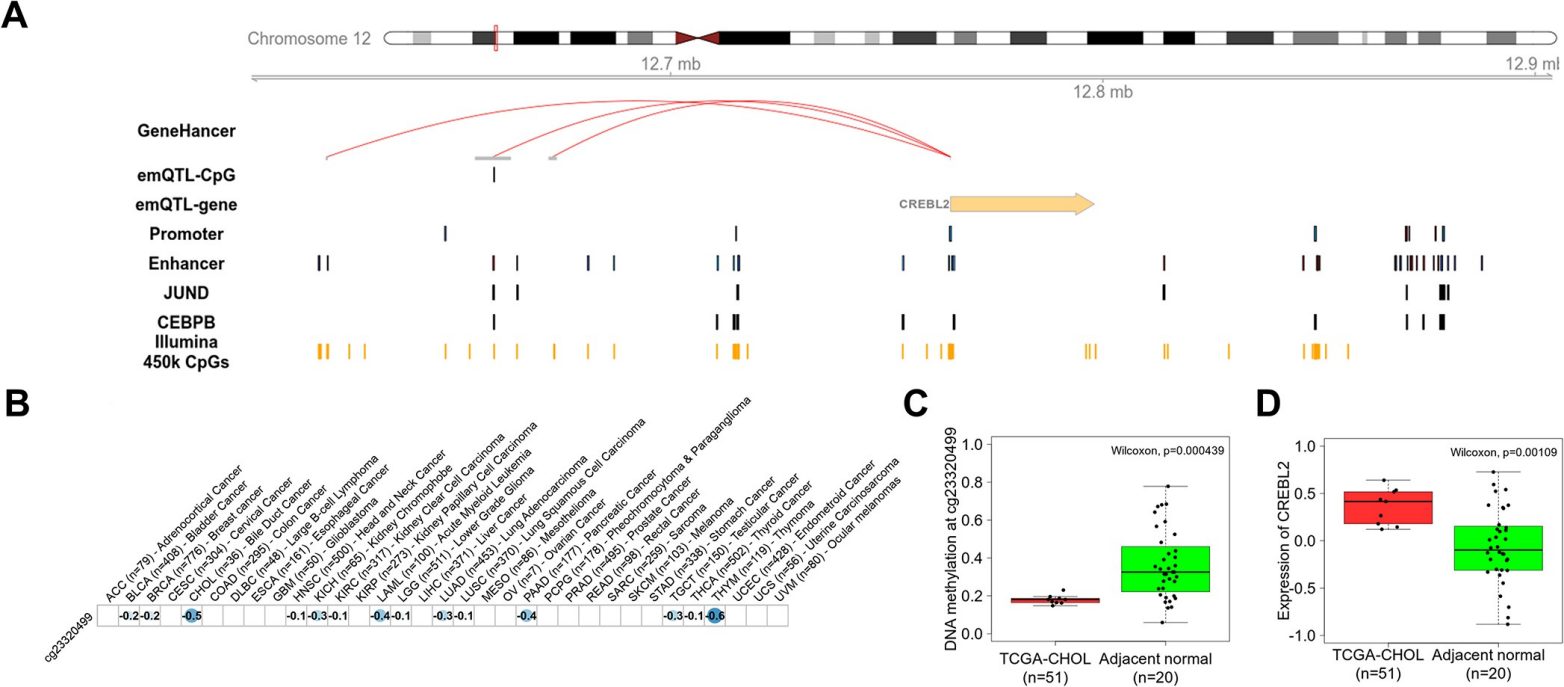
Identification of candidate drivers of proliferative signaling in cancer through knowledge-guided emQTL analysis. (A) Example of a cancer-promoting emQTL identified by knowledge-guided emQTL. The emQTL-CpG (cg23320499) is found in an Encode-defined enhancer region in the binding region of JUND and CEBPB and can form interactions with the CREBL2 promoter. (B) Correlation between DNA methylation at cg23320499 and expression of CREBL2 in each of the 33 cancer types in the TCGA-PANCAN dataset. A blue color indicates a negative correlation, and a red color indicates a positive correlation. The size of the dot represents the strength of the correlation. The Pearson correlation coefficient r is denoted in each box of the dot plot. Only significant correlations after BH-correction are represented with a dot. Box plots showing the difference in DNA methylation at cg23320499 (C) and expression of CREBL2 (D) in bile duct cancer (CHOL) and normal adjacent tissue. Wilcoxon test p-values are denoted.

Since emQTL can be both in cis and trans, and some might rather be passenger emQTL rather than cancer-driving emQTL, we sought to perform emQTL analysis in a knowledge-guided manner to identify candidate emQTL facilitating cancer-promoting functions through direct regulation. By knowing the characteristics of the transcriptional networks linked to the regulation of the cancer-related emQTL communities (i.e., the hormone-like, cell cycle, and metabolism communities), we can perform a knowledge-guided analysis by applying several criteria to the selection of emQTL based on the characteristics of the communities (see methods). Using the knowledge-guided emQTL approach, we identified 1770 proliferation-promoting, 1762 metabolism-promoting, and 1062 hormone-signaling-promoting candidate emQTL in cancer (S1H Table). After identifying candidate emQTL we assessed the link between DNA methylation at the candidate emQTL-CpGs and expression of their linked emQTL-gene for each cancer type and found most candidate emQTL to display correlations within several cancer types (S1H Table). One example of a proliferation-promoting candidate emQTL involves cg23320499 and CAMP Responsive Element Binding Protein Like 2 (CREBL2) found in the GO cell cycle gene set (Fig 5A). CREBL2 associates with the emQTL-CpG cg23320499 and is located in a predicted enhancer in the binding site of JUND and CEBPB. Moreover, DNA methylation at cg23320499 was negatively correlated with the expression of CREBL2 in several cancer types such as thymoma (p-value = 8.71e-11, r-value = -0.6) and bile duct cancer (Fig 5B; p-value = 1.54e-02, r-value = -0.5). In bile duct tumors, a significantly lower methylation level at cg23320499 and higher expression of CRBEL2 were observed compared to normal adjacent tissue (Fig 5C and 5D, respectively). These results suggest that we can identify promising candidate proliferation-promoting emQTL using the knowledge-guided emQTL approach.

### Assessing the role of the cancer-related emQTL in different cancer types

Next, we sought to study the role of the pan-cancer emQTL of the cancer-related communities (i.e., cell cycle, metabolism, and hormone-like communities) in further detail in a cancer-specific manner as case examples to show the link between DNA methylation at the emQTL-CpGs and target gene expression.

#### The cell cycle community

**Fig 6:**
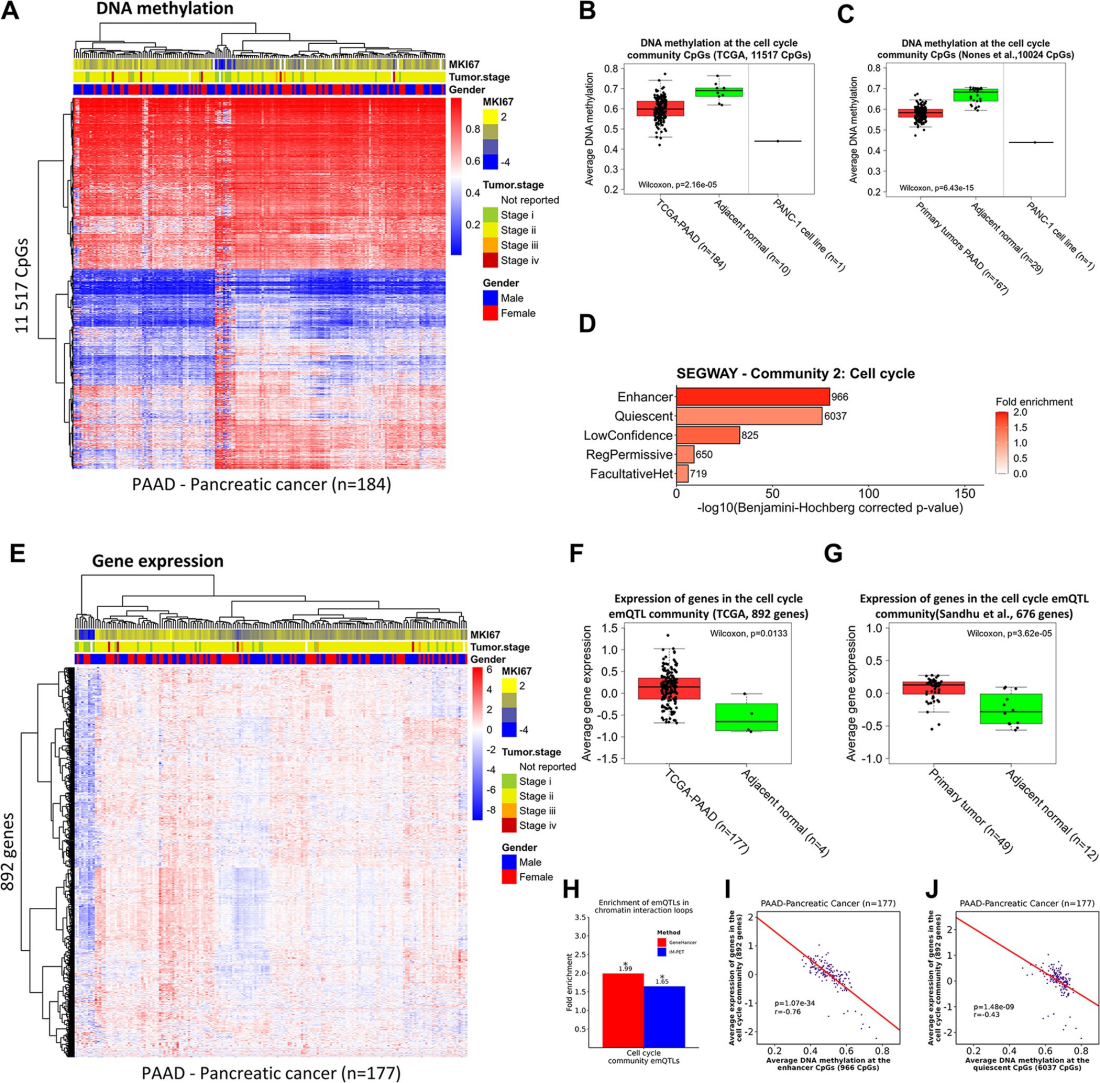
The cell cycle community in pancreatic cancer. (A) Unsupervised hierarchical clustering of DNA methylation levels at the cell cycle community CpGs in pancreatic cancer in TCGA (n = 184). Histopathological features such as tumor stage at diagnosis, MKI67 expression (a marker of proliferation), and gender are annotated in column. (B-C) Box plots showing the average DNA methylation levels at the cell cycle community CpGs in pancreatic cancer tumors from the TCGA cohort and PDAC data obtained from an independent cohort of PDAC tumors (Nones et al. [23]) respectively. Wilcoxon test p-values are denoted. DNA methylation levels in the PANC-1 pancreatic cancer cell line are also included. (C) Bar plots showing the enrichment of the cell cycle community CpGs in Segway-defined regulatory regions from PANC-1. The X-axis shows the log-transformed p-values obtained by hypergeometric testing using the 450k CpGs as background. (E) Hierarchical clustering of the cell cycle community gene expression levels in pancreatic cancer samples from TCGA (n = 177). Histopathological features such as tumor stage at diagnosis, MKI67 expression, and gender are annotated. (F-G) Box plots showing the expression levels of the cell cycle community genes in pancreatic cancer in tumors from TCGA and Sandhu et al. respectively [24]. Wilcoxon test p-values are denoted. (H) Bar plot showing the enrichment of the emQTLs in IM-PET-defined and GeneHancer-defined loops. Statistically significant enrichments are denoted with an asterisk. (I-J) Scatterplot showing the correlation between enhancer methylation (I) and quiescent CpG methylation (J) at the cell cycle bicluster CpGs and the expression of the cell cycle community genes (TCGA-PANCAN, n = 177). The Pearson correlation coefficient and the p-value are denoted.

Among the cancer types, pancreatic tumors exhibited the strongest negative correlation between methylation at the CpGs and expression of the genes in the cell cycle community, as demonstrated in Fig 1D. Due to the aggressive nature of these tumors and the limited treatment options available, we chose to deepen our understanding of the disease by further investigating the role of the pan-cancer emQTLs in the cell cycle community in this particular cancer type. By clustering the DNA methylation levels at the CpGs in the cell cycle community in pancreatic cancer tumors from TCGA, we found a prominent variability in DNA methylation profiles across the tumors (Fig 6A). The CpGs were significantly less methylated in pancreatic cancer tumors compared to normal adjacent tissue both in the TCGA dataset and in the independent cohort from pancreatic ductal adenocarcinomas (PDAC) obtained from Nones et al. (Fig 6B and 6C; p-value = 2.16e-05 and 6.43e-15 respectively) [23]. To investigate the functional role of the cell cycle community CpGs in pancreatic cancer specifically, we obtained epigenome annotation data from Segway derived from multiple ChIP-seq experiments of histone modifications from the pancreatic cancer cell line PANC-1 [32, 70]. Our analysis revealed a statistically significant enrichment of the CpGs in enhancer regions (p-value = 1.77e-80; Fig 6D and S1I Table). We next performed hierarchical clustering of the gene expression profiles of the cell cycle community genes and observed variability between tumors, with some tumors showing consistently higher expression of these genes (Fig 6E). The cell cycle community genes were significantly more expressed in pancreatic tumors compared to normal tissue obtained from TCGA and an independent cohort with expression data obtained from Sandhu et al. [24] (Fig 6F and 6G respectively). The emQTLs in the cell cycle community was enriched in interaction loops in both GeneHancer and the IM-PET data from the pancreatic cancer cell line PANC-1 (Fig 6H and S1G Table), further suggesting a putative regulatory role of altered methylation in pancreatic cancer. Interestingly, in addition to enhancers, CpGs were also enriched in quiescent regions (p-value = 2.31e-76). We hypothesized that enhancer CpGs would have a stronger effect on gene expression, and we thus tested average enhancer methylation and average quiescent-CpG methylation against average gene expression, and indeed observed that the enhancer CpGs showed stronger correlation to gene expression (Fig 6I and 6J; r = -0.76 vs r = -0.43). These findings suggest that loss of enhancer methylation at TFBRs of Fos and Jun family TFs is linked to the upregulation of proliferation-related genes in several cancer types including pancreatic cancer.

#### The metabolism community

Among the cancer types with a correlation between DNA methylation at CpGs and expression of genes in the metabolism community was liver cancer. Moreover, this cancer type exhibited the lowest DNA methylation at the CpGs and expression of the genes in the metabolism community. Moreover, the CpGs in this community were significantly less methylated at the CpGs, and the genes were significantly expressed in this cancer type compared to normal tissue for this community. Therefore, we sought to investigate the role of the pan-cancer emQTLs in the metabolism community in further detail in this cancer type. By performing unsupervised hierarchical clustering of the DNA methylation levels of the CpGs and expression levels of the genes in the metabolism community, we observed clear heterogeneity in the expression profiles and DNA methylation profiles in liver cancer (S18A and S18B Fig respectively). According to the methylation data from TCGA, LICH tumors were significantly less methylated compared to normal adjacent tissue (Wilcoxon p-value = 1.45e-10). They resembled methylation levels close to the purified HepG2 liver cancer cell line (S18C Fig). Similarly, the expression levels differed between liver cancer and normal adjacent tissue in which liver cancer had significantly lower expression levels of the genes in the metabolism community (S18D Fig, Wilcoxon p-value = 2.55e-24). To assess in a cancer-specific manner whether the CpGs in the metabolism community were found in active chromatin, we obtained genome annotation data from Segway from the HepG2 cell line [71]. We found the CpGs in the metabolism community to be significantly enriched in regions carrying active enhancer and promoter chromatin marks. Next, we sought to investigate whether the emQTL in the metabolism community was enriched in predicted chromatin loops specific for a liver cancer cell line, and we found the emQTL in the metabolism community to be significantly enriched in IM-PET interaction loops (p-value = 2.75e-45; S1G Table). Altogether, our findings suggest that DNA methylation at enhancer and promoter regions regulating genes within the metabolism community is an important regulator of metabolism in several cancer types including liver cancer.

#### The hormone-like community

The cancer-type with the strongest negative median correlation coefficient between DNA methylation at the CpGs and expression of genes in the hormone-like community was pancreatic cancer. We continued to study the hormone-like community of this cancer type in further detail. Having found that the CpGs in the hormone-like community were enriched in the TFBR of TFs related to hormone-signaling, we further performed unsupervised hierarchical clustering of the DNA methylation levels at the CpGs and expression of the genes in the hormone-like community in pancreatic cancer. The DNA methylation profiles and expression profiles differed between the pancreatic tumor samples (S19A and S19B Fig). We found the CpGs in the hormone-like community to be significantly less methylated in tumor samples compared to normal adjacent tissue in two independent cohorts (S19C and S19D Fig; p-value = 0.0278 and 1.27e-09 in TCGA and expression data obtained from Nones et al. [23] respectively). Moreover, the genes were significantly upregulated in TCGA and in a pancreatic cancer dataset obtained from Sandhu et al. [24] (p-value = 0.00984 and 3.62e-05 respectively; S19E and S19F Fig). To assess the functional role of the CpGs in pancreatic cancer specifically with regard to histone modifications and regulatory regions, we performed an enrichment analysis using Segway-defined genome annotation data obtained from the PANC-1 [32, 70]. Our analysis showed significant enrichment of the hormone-related CpGs in enhancer and promoter regions in pancreatic cancer (S19G Fig; p-value = 1.44e-165 and 3.29e-25 respectively; S1I Table). Significant enrichment in IM-PET loops for the pancreatic cancer cell line PANC-1 was observed (S19H Fig; p-value = 1.61e-65; S1G Table). Taken together, these results suggest that the depletion of enhancer methylation at CpG sites in the hormone-like community may have a role in regulating transcriptional networks linked to hormone-signaling in several cancer types including pancreatic cancer.

## Discussion

Here, we performed for the first time emQTL analysis in a pan-cancer manner to identify disease-driving transcriptional networks dysregulated due to genome-wide alterations of DNA methylation in cancer. Our results show that hallmark features of cancer (proliferation, hormone signaling and metabolism) are potentially under epigenetic control across cancers, involving key transcription factors and facilitated by chromatin loops. We also showed that the six identified communities of CpGs and genes have prognostic value for cancer patients’ survival.

Epigenomic changes are widespread and frequent in most cancer types [72–75]; however, the understanding of the functional role of epigenetic alterations is limited to specific alterations in specific cancer types. Here, we show that loss of methylation at enhancers controlling genes involved in hallmark cancer functions is a characteristic of most cancer types. Since we observe both hypomethylation and increased expression compared to normal tissue, these results suggest a common architecture across cancer types where oncogenic functions are activated by enhancer hypomethylation. Further studies need to be performed to assess the causal effect of altered DNA methylation, such as CRISPR epigenetic editing by deactivated Cas9 (dCas9) fused with the catalytic domain of epigenetic modifiers [76–79]. Using our knowledge-guided emQTL, we can identify top candidate causal epigenetic alterations that are suitable for future functional studies. Since these emQTL represent potential disease-driving epigenetic alterations, our findings also suggest that these candidates can be utilized as promising targets for targeted epigenetic treatment in the future.

The findings from our study also underscore the clinical relevance of aberrantly methylated DNA and gene dysregulation in predicting survival across cancer types. Aberrant methylation patters, particularly in gene regulatory regions such as enhancers and promoters are increasingly recognized as potential diagnostic, prognostic, and therapeutic biomarkers in cancer [80]. The implication of aberrant DNA methylation in predicting survival has previously been highlighted in several cancer types including breast cancer and gastric cancer [81–83]. The translational impact of our findings extends beyond individual cancer types; by identifying robust associations between DNA methylation levels and gene expression across cancer types, our study contributes to the development of biomarkers that can potentially be applied in a clinical setting to enhance survival prediction.

For the cell cycle community, through the integration of ATAC-seq, ChIP-seq, and GeneHancer loop data, we show that loss of DNA methylation at enhancer regions at the TFBR of proliferation-related TFs is linked with increased expression of proliferation-related genes through GeneHancer-inferred interactions in most cancer types. Among the identified proliferation-related TFs were FOSL1/2 and JUN which can form AP-1 dimers previously shown to directly control the expression of cell cycle regulators such as cyclin D1, p53, p21, ARF, and p16 [84]. Pancreatic cancers displayed the strongest emQTL correlations for the cell cycle community. Previous studies have shown that aberrant DNA methylation in pancreatic cancer is associated with aggressive phenotypes [23, 85–87] and that aberrant DNA methylation has been altered in genes related to cell cycle regulation, Wnt/Notch signaling and cell adhesion [23, 88, 89]. Less is known about how the loss of methylation at distal regulatory regions influences pancreatic cancer tumor phenotype and prognosis. Our findings suggest that loss of enhancer methylation contributes to upregulation of proliferation-related genes in pancreatic cancer in particular and other cancer types in general.

The second cancer-related emQTL community was related to hormone signaling in cancer. We previously described a hormone-like community related to estrogen signaling in breast cancer [18]. This study highlighted that DNA methylation plays a key role in regulating estrogen signaling in breast cancer. Our results suggest that DNA methylation might also play a key role in regulating hormone signaling in other cancer types, which we have also observed in lung cancer [20]. We observe a significant loss of DNA methylation at the hormone-like community CpGs with a concomitant upregulation of genes in the hormone-like community in several cancer types. In pancreatic cancer, we observe that the CpGs are found in regions with active chromatin marks in enhancer regions. These enhancers are linked to the hormone-like genes through looping. Moreover, according to the TF enrichment analysis, the CpGs enriched in the binding sites of TFs involved in hormone-signaling such as ESR1 [59] and AR [60]. Activation of AR associated with human carcinogenesis in pancreatic cancer has been well described [90]. Expression of the estrogen receptor in pancreatic adenocarcinoma has previously been detected and has been linked to poor prognosis [91, 92]. Our findings suggest that loss of enhancer methylation can also be a driver of hormone-signaling in several cancer types such as pancreatic cancer.

The third cancer-related emQTL community was related to the regulation of metabolism in cancer. Reprogramming of the cellular metabolism is considered one of the core hallmarks of cancer, but little is still known about the mechanisms that drive this shift in the metabolism [1]. The CpGs were enriched for enhancers and binding regions of TFs involved in metabolism such as the HNF family of TFs [49–51] and RXRA [52]. Moreover, we found the emQTL to be more than expected by chance in GeneHancer and IM-PET loops. We thus hypothesize that loss of DNA methylation at promoter and enhancer regions may play a regulatory role in metabolism in cancer. More studies on the metabolism emQTL community will be of future interest.

We discovered three emQTL communities representing varying degrees of non-cancer cell infiltration. Because infiltrating cells from the microenvironment have both different methylation profiles and expression profiles, varying degrees of infiltration will cause statistical associations between DNA methylation and gene expression. This has been described in breast cancer for the immune signal (immune cells; [18]) and the EMT signal (fibroblast infiltration; [19]), and has also been observed by others [93].

Fibroblasts are prominent cell types of the tumor microenvironment in solid tumors and express the genes found in the hallmark EMT gene set and may induce EMT in epithelial cells [94, 95]. Interestingly, among the genes residing in the EMT community were established fibroblast marker genes including COL1A2, COL5A1, and CD248 [96, 97]. Fibroblast infiltration has previously been linked to poor treatment response in several cancer types and has been found to modulate immune cell activity and promote immune evasion [98]. Moreover, an increasing number of studies have found fibroblast infiltration to promote tumorigenesis through the secretion of cancer-associated fibroblast (CAF) secreted factors such as TGF-β [99]. TGF-β stimulation has been shown to induce changes in the DNA methylation landscape in several cancer types such as ovarian cancer, liver cancer, and prostate cancer [100–102]. Interestingly, the TGFB gene was found residing within the EMT community, suggesting a possible role in cancer carcinogenesis either directly by upregulation in the cancer cells or indirectly through crosstalk between CAFs and tumor cells. Although the EMT community is associated with fibroblast infiltration, we further hypothesize that there could be a less detectable EMT signal in the EMT community from the cancer cells that is masked by fibroblast infiltration in the tumor biopsies. As studies have shown, crosstalk between fibroblasts and cancer cells through CAF-secreted factors can alter the methylation landscape of cancer cells and promote EMT [99–102]. A more detailed study of the EMT community regarding EMT in cancer cells would therefore be of future interest.

In our previous reports, we showed that estrogen signaling and proliferation in breast cancer and hormone signaling and lipid metabolism in lung cancer was linked to loss of enhancer methylation, and using single cell RNA-seq data, we showed that the identified genes were expressed mainly in cancer cells [19, 20]. A limitation of the current work is the lack of a pan-cancer single cell RNA-seq data set, but our previous results validate a subset of the communities for breast and lung cancer.

In this work we use the methylation value of single CpGs (as opposed to regions) when performing the analyses. This has the advantage of identifying individual CpGs (or small regions of CpGs where only one is covered by the array) that may be important for transcription factor binding or regulation of gene expression. A limitation of using single CpGs from array technology such as Illumina HumanMethylation450 is that probes are unevenly distributed across genes potentially leading to a bias (higher probability of identifying genes with more associated probes) as described by Maksimovic et al [103]. In this study, we observed that the genes in the cell cycle, metabolism and hormone-like communities (the most important communities) showed quite similar distribution of gene length compared to all genes genome-wide, which suggest that in this analysis we are not introducing bias in the analysis by using single CpGs.

There might be various confounders when performing genome-wide correlation analyses such as SNPs, CNVs and clinical and demographic variables; for example, germline variation has been reported to affect the epigenome [104]. Recently, a study showed that in whole blood samples concomitant variations in DNA methylation and gene expression between individuals were explained by germline sequence variants [105]; however, this study did not assess hematopoietic development, a process where epigenetics regulation is critical [106–109] and can to a much lesser degree be affected by germline variation. Given the frequency and amplitude of epigenetic alterations in cancer, we consider it likely that the observed variations are predominantly caused by somatic alterations and subtypes of cancer. Further studies into each cancer type and the regulatory role of epigenetic alterations could assess the role of these potential confounders.

## Conclusion

Genome-wide loss of enhancer methylation at specific CpG sites was linked with transcriptional dysregulation of genes related to proliferation, metabolism, and hormone-signaling in cancer. Moreover, we identify key transcription factors involved in regulating each emQTL network. This propagates the idea that the hallmarks of cancer are epigenetically regulated, or at least tightly linked to epigenetic alterations. Our study provides insight into how alterations in DNA methylation in the context of histone modifications and the chromatin 3D structure govern and influence transcriptional regulation in a cancer-specific manner. Our knowledge-guided emQTL approach identifies potential therapeutic targets for future targeted epigenetic treatment and cancer biomarkers.

## Supporting information

S1 Table Summary of data from this study. (A) Overview of the CpGs in the emQTL communities. (B) Overview of the genes in each emQTL community. (C) Enrichment of genes in gene sets from the MSigDB by community. Only gene sets with a significant enrichment after BH-correction are shown. (D) Reactome pathway enrichment analysis using the gene sets obtained from each emQTL community. (E) Enrichment of CpGs in community 1–6 in Encode-defined cis-regulatory elements defined by ENCODE. (F) Enrichment of CpGs in community 1–6 in UniBind-defined TFBRs. Only TFs showing a statistically significant enrichment are shown. (G) Enrichment of emQTL in community 1–6 in GeneHancer-defined chromatin loops and IM-PET loops. (H) Candidate cancer-promoting emQTL related to the regulation of metabolism, proliferation, and hormone-signaling. p-values and r-values obtained from Pearson correlation between DNA methylation at CpGs and expression levels of genes are included for the discovery and validation dataset from the TCGA-PANCAN dataset. R-values obtained from the correlation analysis in the 33 different TCGA-PANCAN cancer types independently are annotated to indicate if the correlation was significant after BH correction. (I) Enrichment emQTL -CpGs in Segway-defined regulatory regions in the PANC-1 and HepG2 cell lines by community.
(XLSX)

S1 Fig Overview of the pan-cancer emQTL pipeline. Assessment of the link between DNA methylation and gene expression was performed by Pearson correlation. The significant correlations found in the discovery subset of the TCGA-PANCAN dataset (n = 4104) were then validated in the TCGA-PANCAN validation dataset (n = 4089).
(PNG)

S2 Fig Overview of the pan-cancer emQTL. Density plot showing the distribution of the Pearson correlation coefficients in the discovery (A) and validation (B) datasets from the TCGA-PANCAN dataset for all the 1 192 567 pan-cancer emQTL. (C) Bar plot showing the number of pan-cancer emQTL showing negative and positive correlations between DNA methylation and gene expression.
(PNG)

S3 Fig Reactome pathway enrichment analysis. Pathway enrichment analysis results using the genes from each emQTL community as input. The length of the bar represents the -log10 FDR-corrected p-value. Only the top 10 most significantly enriched pathways are shown. All bars crossing the dotted line represents significantly enriched pathways.
(PNG)

S4 Fig Distribution of CpGs mapping to all genes and genes of the identified clusters. (PNG)

S5 Fig Overview of the TFBR overlap between the top enriched TFs in each emQTL community represented as Upset plots. (PNG)

S6 Fig Enrichment of emQTL in chromatin loops. Bar plot showing the enrichment of pan-cancer emQTL from each emQTL community in GeneHancer, IM-PET loops (PANC-1, HepG2, A549, HCC1954, MCF7, HCT116, HELA, and K562), and ChIA-PET Pol2 loops (MCF7). The height of the bars represents the -log10(BH-corrected p-values) obtained by hypergeometric testing. Statistically significant enrichments are marked with an asterisk (BH-corrected p-value<0.05).
(PNG)

S7 Fig Survival prediction of the pan-cancer emQTL. (A) The pan-cancer patients were randomly split into 80% training and 20% test data 50 times. Each box represents Uno’s C-indexes estimated from the randomly split 20% test data. Due to few samples for ovarian cancer (n = 8) and high censoring in prostate- and testicular cancer, these cancer types were not included in the analysis.
(PNG)

S8 Fig DNA methylation at the TFBR in the cell cycle, metabolism, and hormone-like emQTL community. Box plots showing the average DNA methylation at the CpGs in the TFBR of some of the top enriched TFs in the cell cycle (A), metabolism (B), and hormone-like (C) communities. DNA methylation levels from available normal samples are included. BH-corrected Wilcoxon-test p-values are denoted.
(PNG)

S9 Fig DNA methylation at CpGs in the hormone-like and metabolism communities. Heatmaps showing the DNA methylation levels of the hormone-like (A) and metabolism (B) communities CpGs in the TCGA-PANCAN dataset (n = 8229) following unsupervised hierarchical clustering of DNA methylation levels. Rows represent CpGs and tumor samples represent the columns. Unmethylated and methylated CpGs are shown in blue and red points respectively. Histopathological features including cancer type, organ system and histology are indicated in the columns. Box plots showing the average DNA methylation levels at the hormone-like (C) and metabolism (D) communities CpGs by TCGA cancer type. DNA methylation levels from available normal samples are included. BH-corrected Wilcoxon-test p-values are denoted. (C) Box plots showing the accessibility of the hormone-like (E) and metabolism (F) communities CpGs for different TCGA-PANCAN cancer types obtained by ATAC-seq. A higher value represents less compact chromatin and vice versa.
(PNG)

S10 Fig Expression of genes in the hormone-like and metabolism communities. Hierarchical clustering of the expression levels of the genes in the hormone-like (A) and metabolism (B) communities in the TCGA-PANCAN dataset (n = 9875). Rows represent genes and column tumor samples. Red points indicate high expression and blue points indicate low expression. Histopathological features including organ system, cancer type and histology are included. Box plots showing the average expression levels of the hormone-like (C) and metabolism (D) community genes by cancer type. Tumor samples with normal tissue are included. BH-corrected Wilcoxon-test p-values are denoted. Chromatin accessibility around the hormone-like (E) and metabolism (F) community genes in the TCGA-PANCAN tumor samples.
(PNG)

S11 Fig Association between DNA methylation and expression versus tumor purity. Scatter plots showing the correlation between DNA methylation at the emQTL-CpGs and expression of emQTL genes versus the ASCAT tumor purity estimates in the TCGA-PANCAN dataset. P-values and Pearson correlation coefficients are denoted.
(PNG)

S12 Fig DNA methylation at CpGs and expression of genes in the immune community. (A) Unsupervised hierarchical clustering of DNA methylation levels at the CpGs in the immune community in TCGA (n = 8229). The red points indicate methylated CpGs and the blue points indicate unmethylated. Rows represent CpGs and columns represent tumor samples. (B) Box plot showing the average DNA methylation levels at the immune community CpGs by TCGA cancer type. DNA methylation levels from available normal samples are included. BH-corrected Wilcoxon-test p-values are denoted. (C) Unsupervised hierarchical clustering of gene expression levels of the immune community genes in each TCGA-PANCAN dataset (n = 9875). Rows represent genes and columns represent tumor samples. Red points indicate high expression and blue points low expression. Histopathological features including organ system, cancer type and histology are included. (D) Box plot showing the average expression levels of the immune community genes by cancer types. Tumor samples with normal tissue are included. BH-corrected Wilcoxon-test p-values are denoted.
(PNG)

S13 Fig emQTL community 5 is linked to immune infiltration. (A) Box plot showing the link between DNA methylation at the immune community CpGs and immune cell infiltration. The level of immune infiltration in tumor samples from TCGA was determined using the xCell deconvolution tool [36]. Tumor samples were divided into quartile groups based on the severity of immune infiltration; low, moderate, high, and severe. DNA methylation levels at the immune community CpGs in isolated immune cells (T-cells, monocytes, B-cells, and Leukocytes) and cancer cell lines from the NCI-60 cancer cell lines are included. (B) Expression of the immune community genes in relation to the xCell-derived immune score. (C) Box plot showing the association between the xCell-derived immune score and tumor purity estimates obtained by ASCAT. Kruskal-Wallis test p-values determined by comparing the quartile groups are denoted in each box plot (A-C).
(PNG)

S14 Fig DNA methylation at CpGs and expression of genes in the EMT community. (A) Unsupervised hierarchical clustering of DNA methylation levels at the EMT community CpGs in TCGA-PANCAN (n = 8229). Red points indicate methylated CpGs and blue points indicate unmethylated. Rows represent CpGs and columns represent tumor samples. (B) Box plot showing the average DNA methylation levels at the EMT community CpGs by cancer type. DNA methylation levels from available normal samples are included. BH-corrected Wilcoxon-test p-values are denoted. (C) Unsupervised hierarchical clustering of gene expression levels of the EMT community genes in each TCGA-PANCAN dataset (n = 9875). Rows represent genes and column tumor samples. Red points indicate high expression and blue points indicate low expression. Histopathological features including organ system, cancer type and histology are included. (D) Box plot showing the average expression levels of the EMT community genes by cancer types. Tumor samples with normal tissue are included. BH-corrected Wilcoxon-test p-values are denoted.
(PNG)

S15 Fig emQTL community 6 is associated with fibroblast infiltration. (A) Box plot showing the association between expression of the EMT community genes in relation to fibroblast infiltration. Fibroblast infiltration level in the tumor samples in TCGA was determined by the xCell deconvolution tool. Tumor samples were divided into quartile groups based on the severity of fibroblast infiltration; low, moderate, high, and severe. (B) The association between fibroblast infiltration and DNA methylation at the EMT community CpGs. DNA methylation levels obtained from human mammary fibroblasts and the NCI-60 cancer cell lines are also included. (C) shows the association between fibroblast infiltration and tumor purity estimates from ASCAT. Kruskal-Wallis test p-values comparing the quartile groups are denoted in each plot.
(PNG)

S16 Fig DNA methylation at CpGs and expression of genes in the NE-like community. (A) Hierarchical clustering of DNA methylation levels at the NE-like community CpGs in the TCGA-PANCAN dataset (n = 8229). Rows represent CpGs and columns represent tumor samples. Methylated and unmethylated CpGs are shown as red and blue dots respectively. Cancer type, organ system and histology of the cancer type are indicated. (B) Heatmap showing the expression levels of the NE-community genes in the TCGA-PANCAN dataset (n = 9875). Rows and columns represent genes and samples respectively and were ordered by unsupervised hierarchical clustering. Blue points indicate low expression while red points indicate high. The samples are annotated by cancer type, organ system and histology. (C) Box plot showing the average DNA methylation levels at the NE-like community CpGs by cancer type. DNA methylation levels from normal samples are included for those cancer types with data available. BH-corrected Wilcoxon-test p-values are denoted. (D) Box plot showing the average expression of genes in the NE-like community by cancer type. BH-corrected Wilcoxon test p-values are denoted by comparing expression levels of genes between tumor and normal samples. (E) Boxplot showing the chromatin accessibility around the CpGs in the NE-like community as determined by ATAC-seq of the tumor samples from TCGA-PANCAN. A high value represents more open chromatin while a low value represents more compact chromatin. The chromatin accessibility around the NE-like community genes is visualized in (F) for the TCGA-PANCAN dataset.
(PNG)

S17 Fig Community 3 is linked to the infiltration of non-tumor cells. Boxplots showing the expression of the NE-like community genes in the non-neuron (NN) related cancer types (A) and the neuron-related cancer types (LGG, PCPG, GBM; D) in TCGA. The tumors were divided into four quartile groups (Low, moderate, high, and severe) based on the severity of infiltration using the microenvironment score obtained from xCell. The DNA methylation levels at the NE-like community CpGs in NN and non-NN tumors are shown in (B) and (E) respectively. (C) and (F) shows the associations between the microenvironment score and the ASCAT tumor purity score in the non-NN and NN tumors respectively. Kruskal-Wallis test p-values are denoted in each box plot.
(PNG)

S18 Fig DNA methylation at CpGs and expression of genes in the metabolism community in liver cancer. (A) Heatmap showing the level of DNA at the metabolism community CpGs in the TCGA-LIHC dataset (n = 447). Red point indicates methylated CpGs and blue points indicate unmethylated CpGs. Rows represent CpGs and columns represent tumor samples. (B) Unsupervised hierarchical clustering of expression levels of genes in the metabolism community in liver cancer (n = 377). Rows represent genes and columns represent tumor samples. (C) DNA methylation at the CpGs in the metabolism community in liver cancer (TCGA-LIHC). Box plot showing the expression of genes in the metabolism community in liver cancer (TCGA-LIHC). Wilcoxon-rank sum test p-values between tumor and normal samples are denoted in C and D. (E) Enrichment of the metabolism community CpGs in LIHC-defined regulatory regions obtained by Segway. The length of the bars represents -log10-transformed p-values obtained by hypergeometric testing using all the 450k probes as background. Only significant enrichments (BH-corrected p-value<0.05) are shown.
(PNG)

S19 Fig emQTL community 4 is associated with hormone-related signaling. (A) Unsupervised hierarchical clustering of the DNA methylation levels at the hormone-like community CpGs in pancreatic cancer (TCGA-PAAD). (B) Hierarchical clustering of the expression levels of the hormone-like community genes in pancreatic cancer (TCGA-PAAD). (E-F) Box plot showing the DNA methylation levels at the CpGs in the cell cycle community in TCGA (C) and the independent pancreatic cancer dataset obtained from Nones et al. [23] (D). DNA methylation levels for the PANC1 cell line are also included. Expression of the hormone-like community genes in pancreatic tumors from TCGA (E) and an independent cohort obtained from Sandhu et al. [24] (F). Wilcoxon-rank sum test p-values are denoted in each box plot. (G) Bar plot showing the enrichment of the hormone-like community CpGs in different regulatory regions of the genome according to SEGWAY from PANC1. (H) Enrichment of emQTL in the hormone-like community in IM-PET chromatin loops from the PANC1 pancreatic cancer cell line. The enrichment was determined using hypergeometric tests of significance using all possible in cis CpG-gene pairs as background. Significant enrichments after BH correction are marked with an asterisk.
(PNG)
