# Supplementary figures and images for: Integrative pan-cancer analysis reveals a common architecture of dysregulated transcriptional networks characterized by loss of enhancer methylation
Source: PLoS Comput Biol. 2024 Nov 18;20(11):e1012565. doi: 10.1371/journal.pcbi.1012565 (PMC11611269; doi:10.1371/journal.pcbi.1012565)

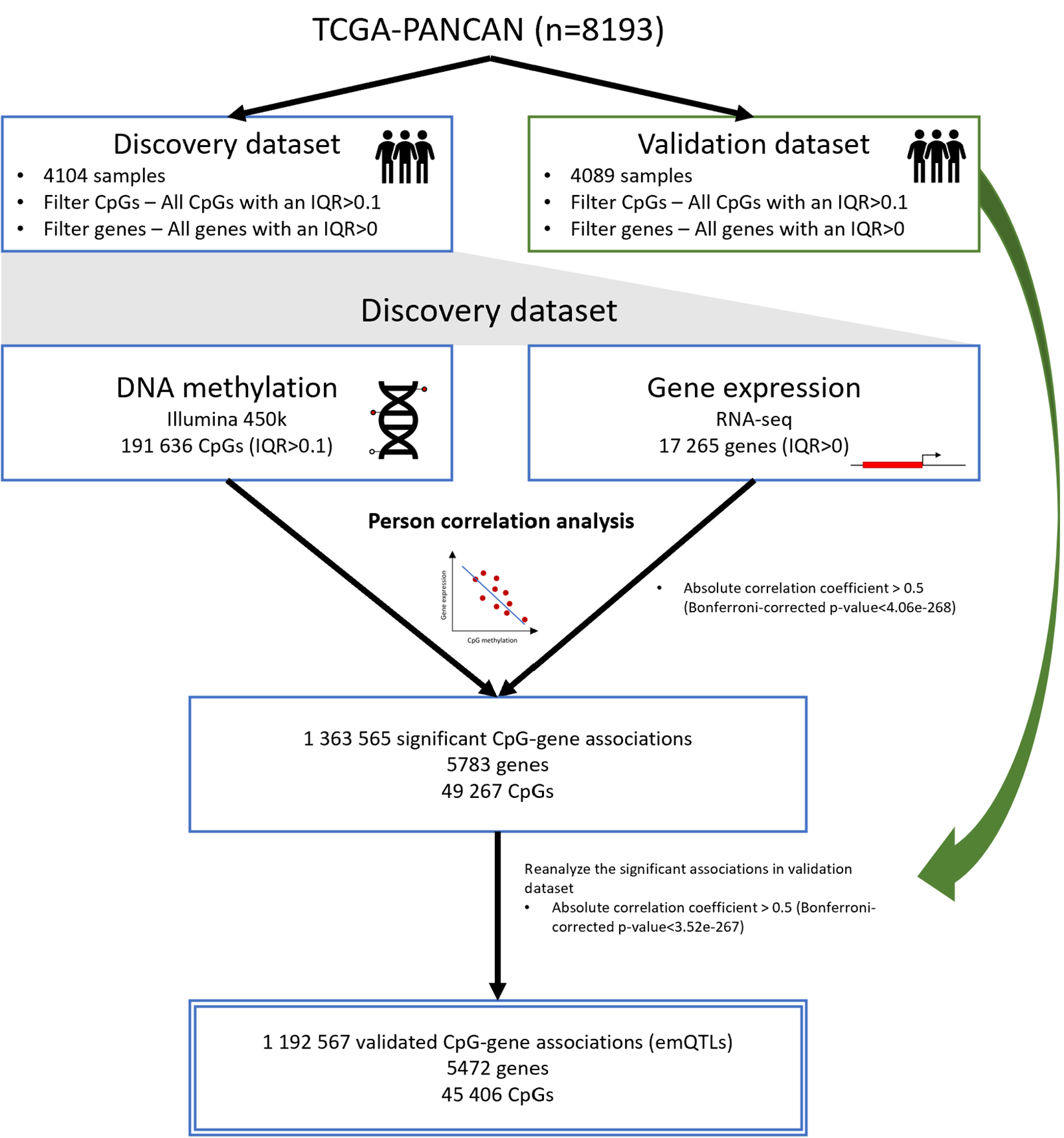

Supplement: S1 Fig — Assessment of the link between DNA methylation and gene expression was performed by Pearson correlation. The significant correlations found in the discovery subset of the TCGA-PANCAN dataset (n = 4104) were then validated in the TCGA-PANCAN validation dataset (n = 4089). (PNG) [file pcbi.1012565.s002.png]

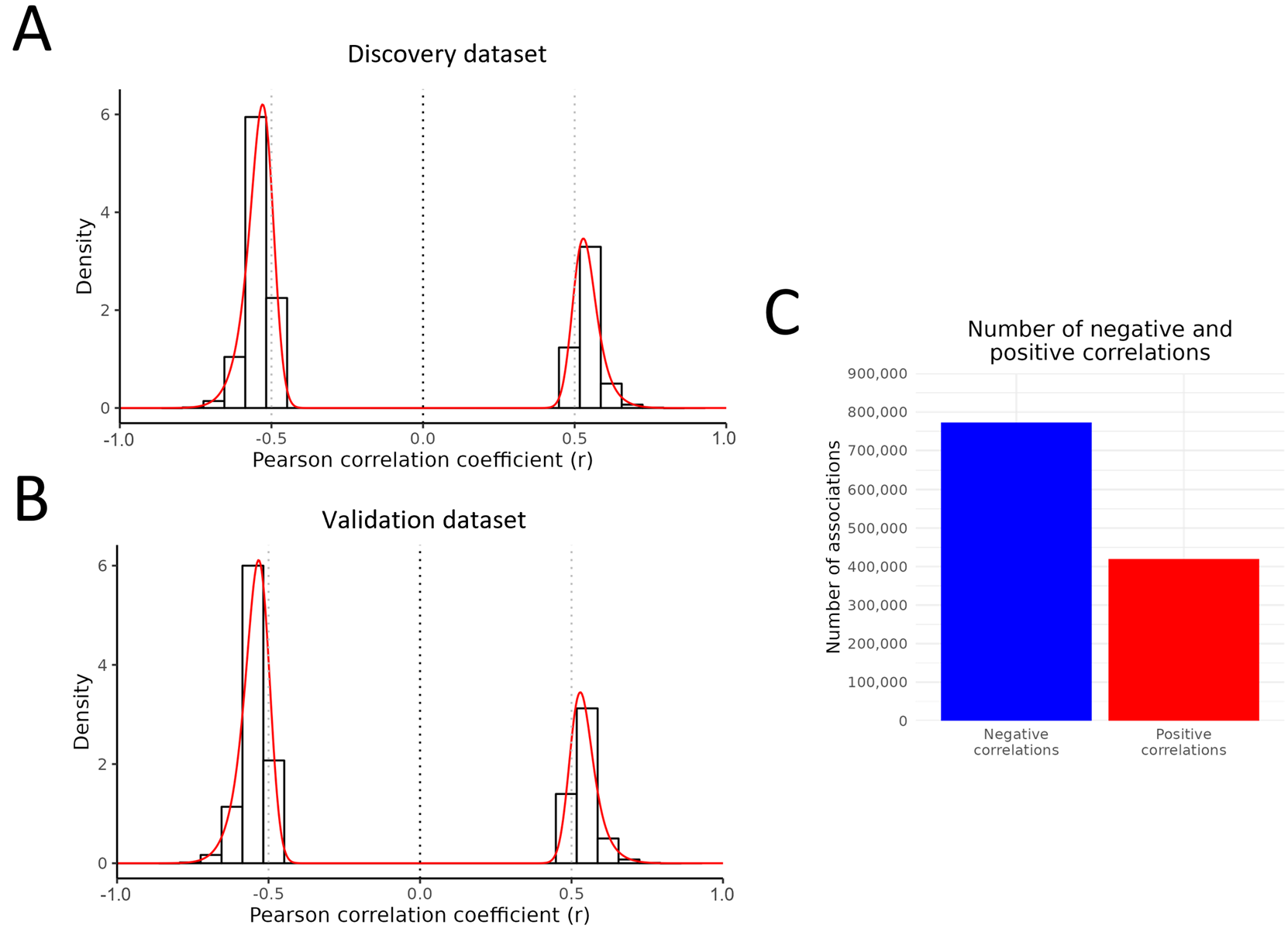

Supplement: S2 Fig — Density plot showing the distribution of the Pearson correlation coefficients in the discovery (A) and validation (B) datasets from the TCGA-PANCAN dataset for all the 1 192 567 pan-cancer emQTL. (C) Bar plot showing the number of pan-cancer emQTL showing negative and positive correlations between DNA methylation and gene expression. (PNG) [file pcbi.1012565.s003.png]

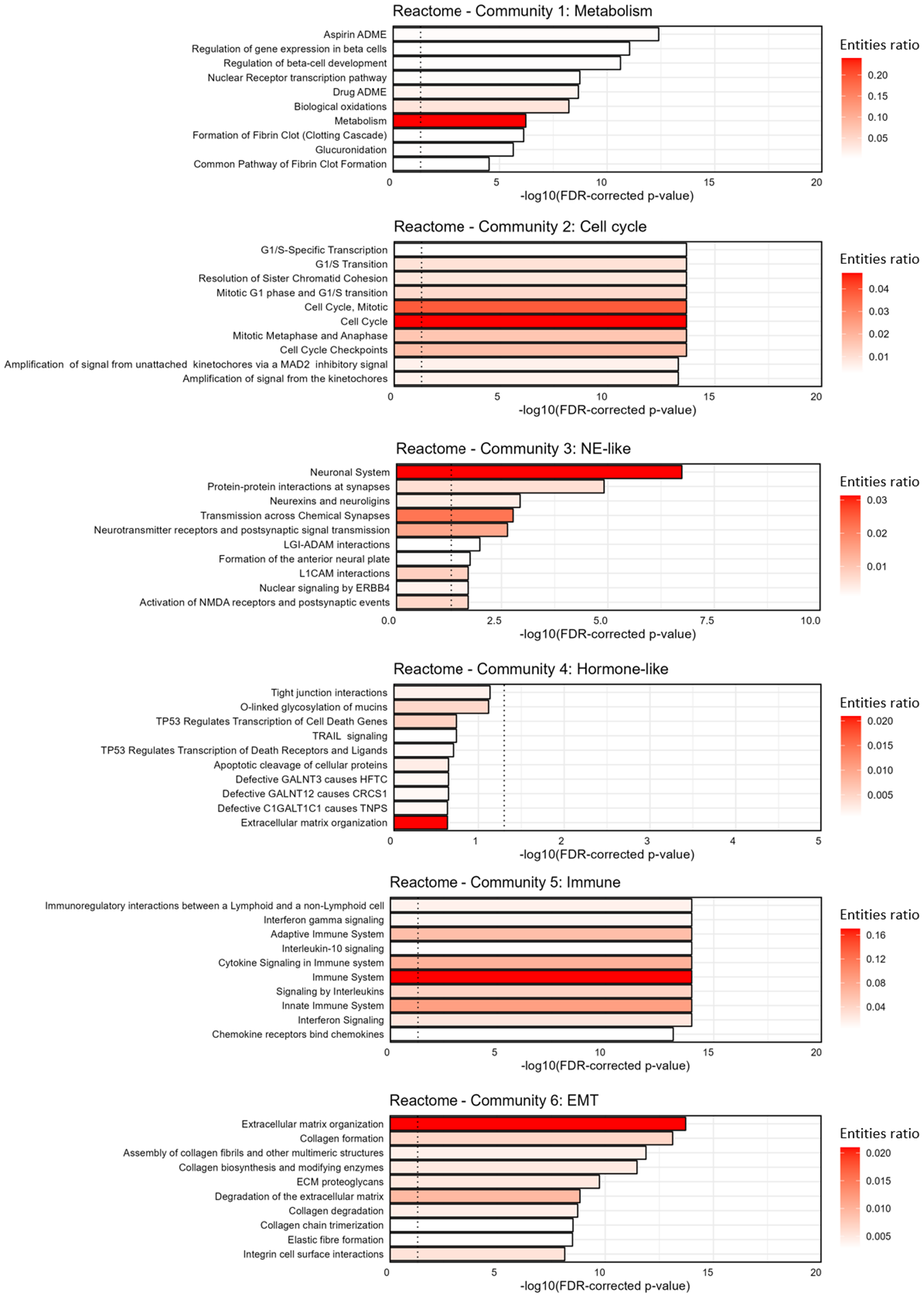

Supplement: S3 Fig — Pathway enrichment analysis results using the genes from each emQTL community as input. The length of the bar represents the -log10 FDR-corrected p-value. Only the top 10 most significantly enriched pathways are shown. All bars crossing the dotted line represents significantly enriched pathways. (PNG) [file pcbi.1012565.s004.png]

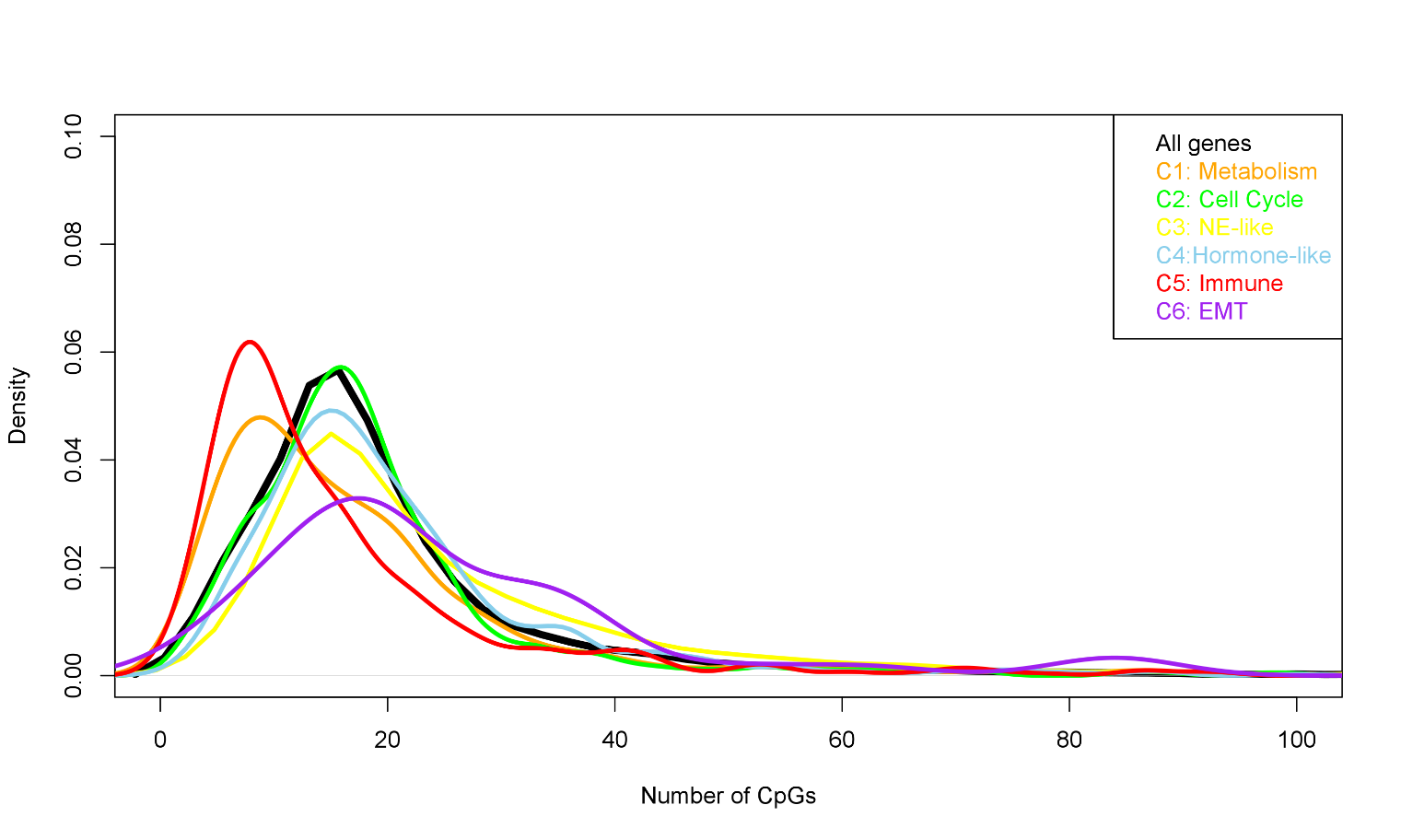

Supplement: S4 Fig — (PNG) [file pcbi.1012565.s005.png]

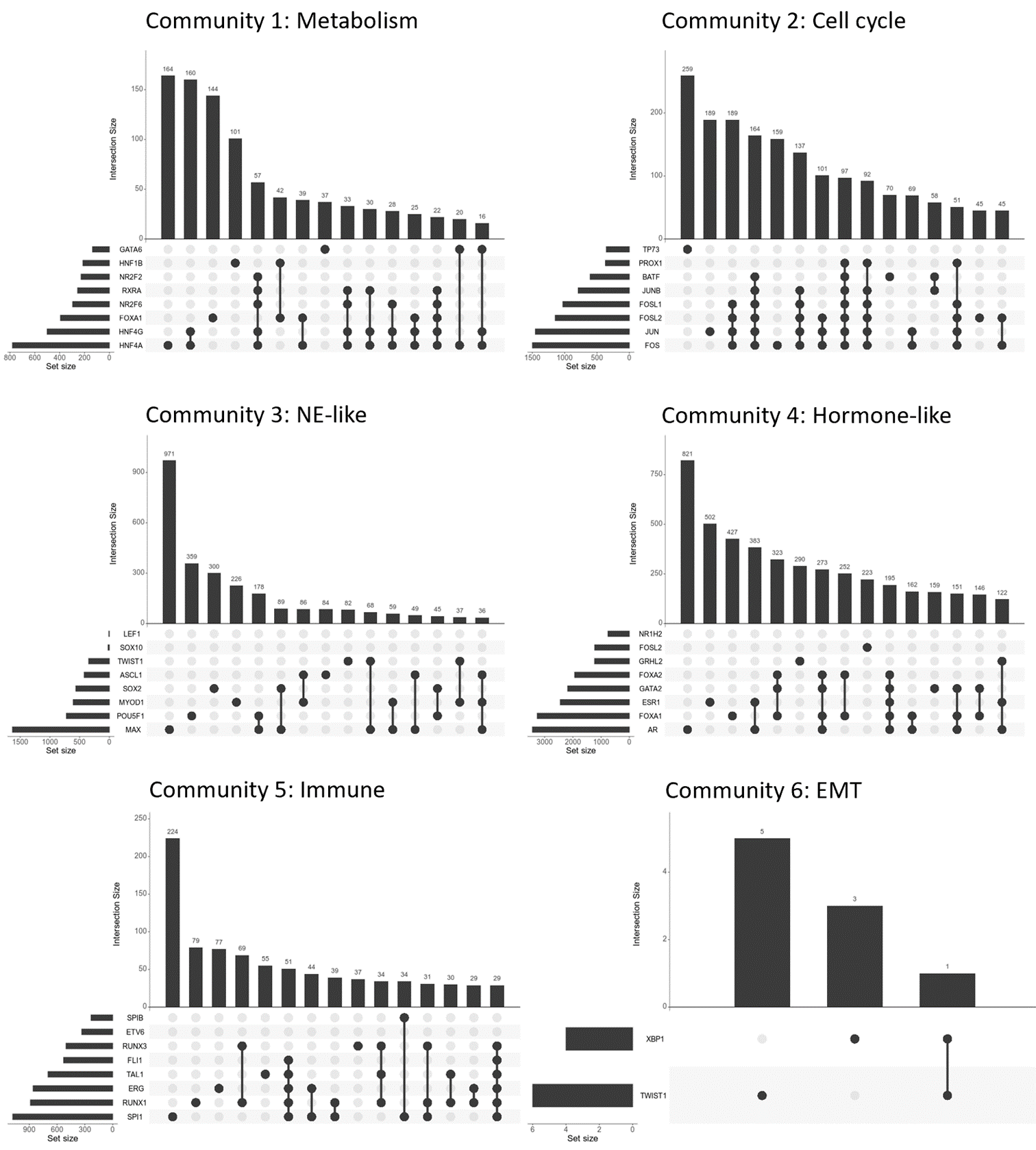

Supplement: S5 Fig — (PNG) [file pcbi.1012565.s006.png]

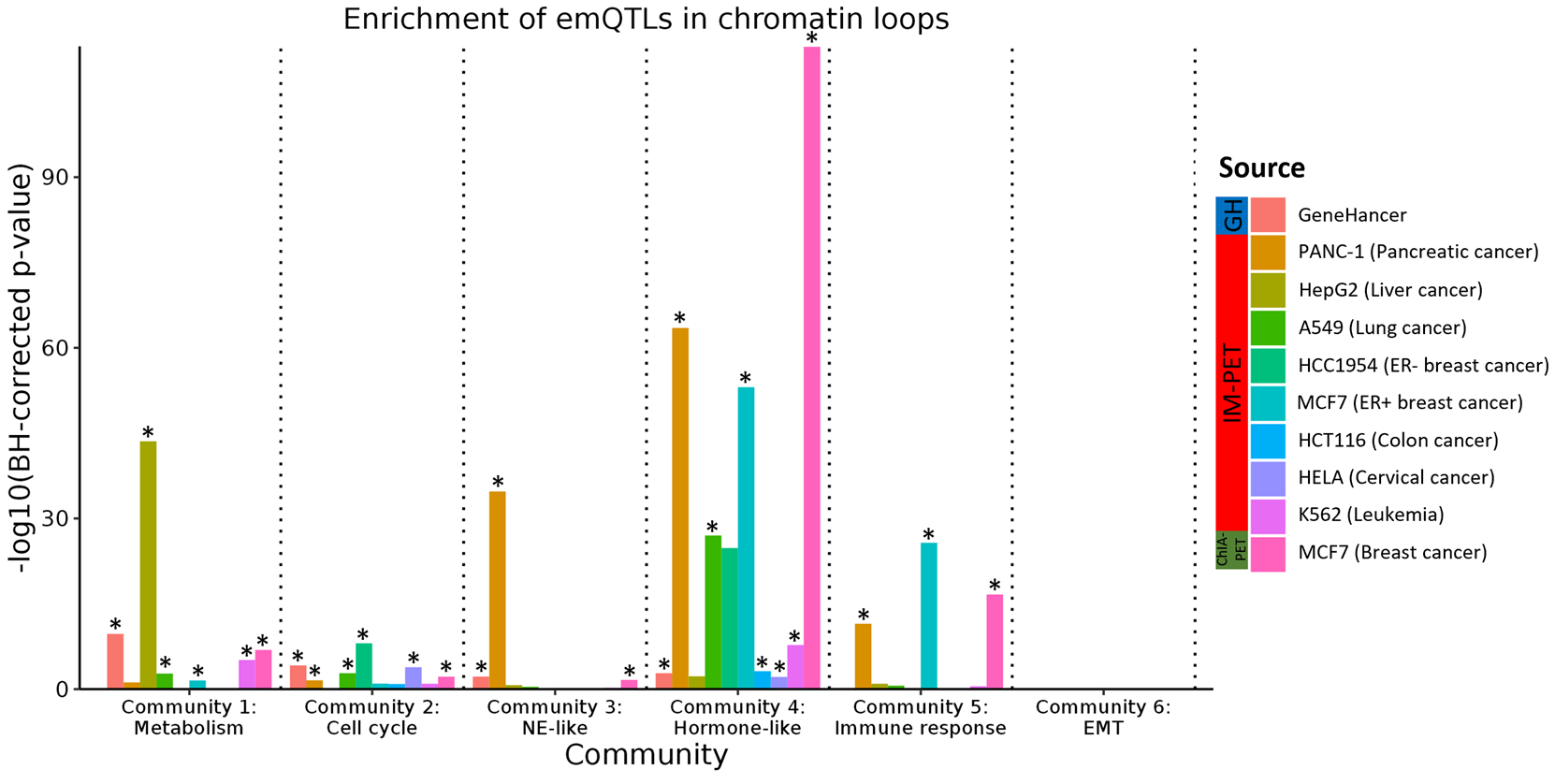

Supplement: S6 Fig — Bar plot showing the enrichment of pan-cancer emQTL from each emQTL community in GeneHancer, IM-PET loops (PANC-1, HepG2, A549, HCC1954, MCF7, HCT116, HELA, and K562), and ChIA-PET Pol2 loops (MCF7). The height of the bars represents the -log10(BH-corrected p-values) obtained by hypergeometric testing. Statistically significant enrichments are marked with an asterisk (BH-corrected p-value<0.05). (PNG) [file pcbi.1012565.s007.png]

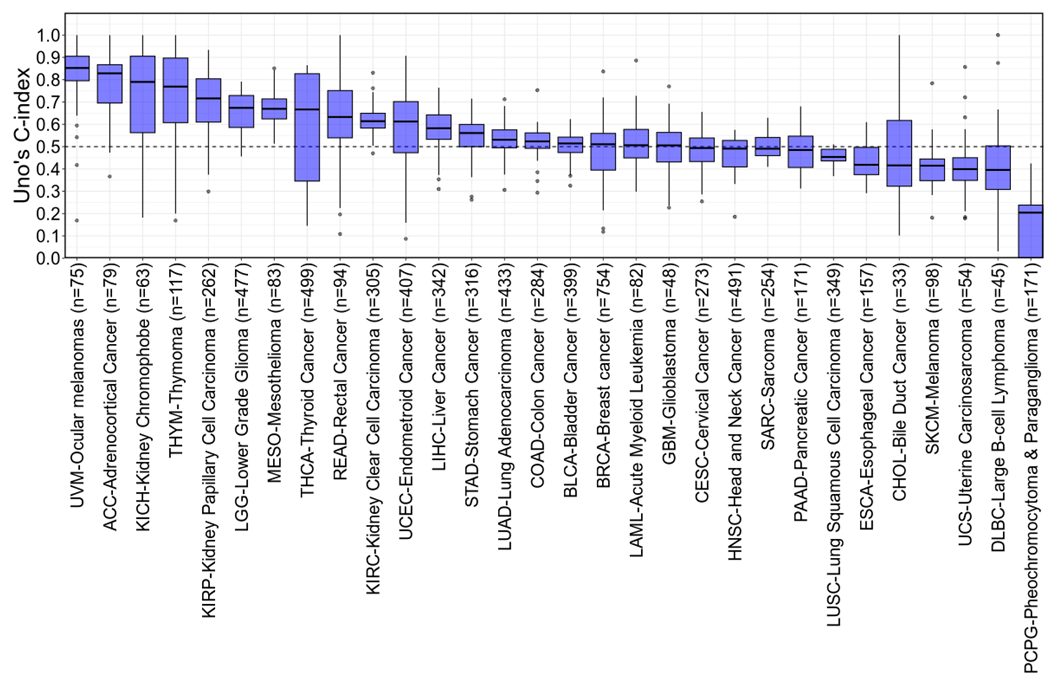

Supplement: S7 Fig — (A) The pan-cancer patients were randomly split into 80% training and 20% test data 50 times. Each box represents Uno’s C-indexes estimated from the randomly split 20% test data. Due to few samples for ovarian cancer (n = 8) and high censoring in prostate- and testicular cancer, these cancer types were not included in the analysis. (PNG) [file pcbi.1012565.s008.png]

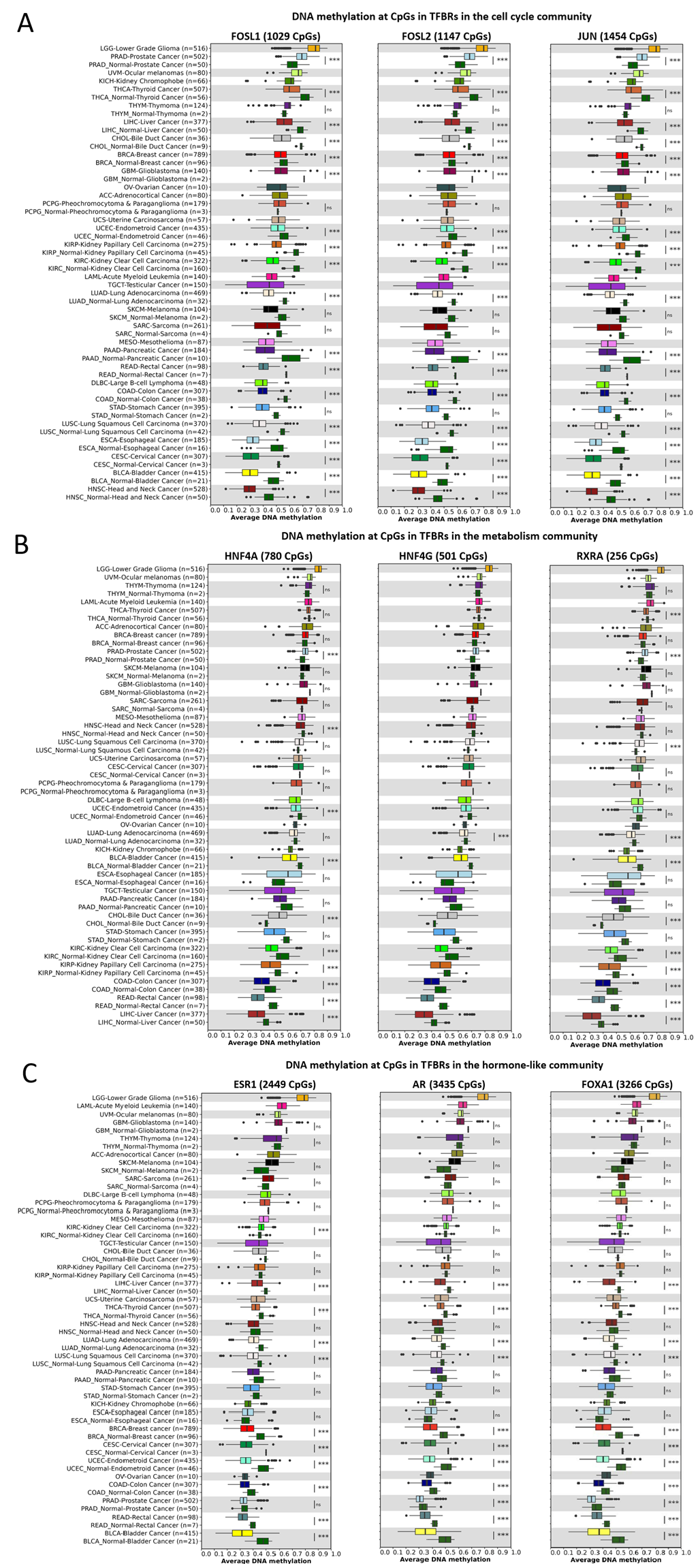

Supplement: S8 Fig — Box plots showing the average DNA methylation at the CpGs in the TFBR of some of the top enriched TFs in the cell cycle (A), metabolism (B), and hormone-like (C) communities. DNA methylation levels from available normal samples are included. BH-corrected Wilcoxon-test p-values are denoted. (PNG) [file pcbi.1012565.s009.png]

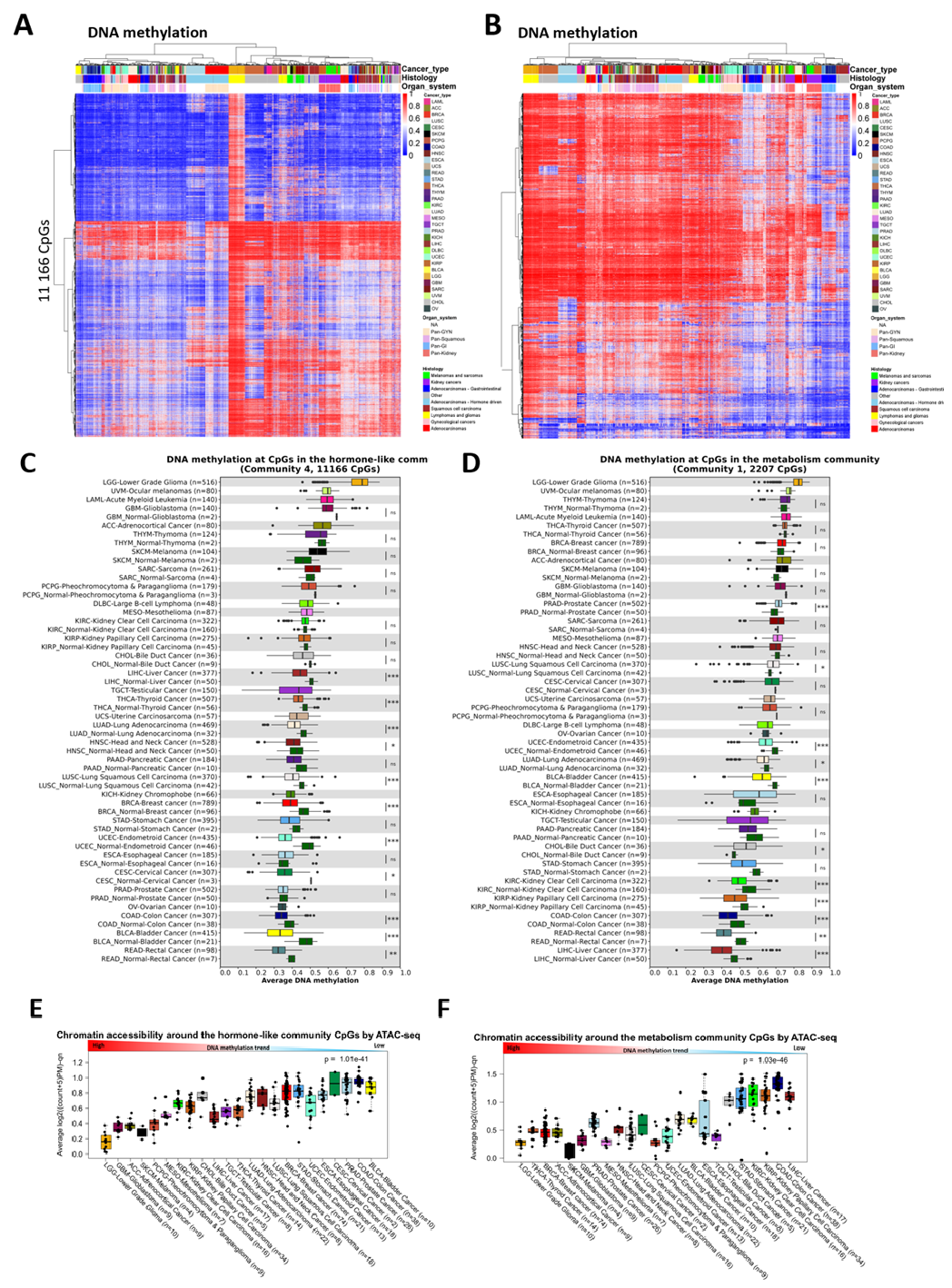

Supplement: S9 Fig — Heatmaps showing the DNA methylation levels of the hormone-like (A) and metabolism (B) communities CpGs in the TCGA-PANCAN dataset (n = 8229) following unsupervised hierarchical clustering of DNA methylation levels. Rows represent CpGs and tumor samples represent the columns. Unmethylated and methylated CpGs are shown in blue and red points respectively. Histopathological features including cancer type, organ system and histology are indicated in the columns. Box plots showing the average DNA methylation levels at the hormone-like (C) and metabolism (D) communities CpGs by TCGA cancer type. DNA methylation levels from available normal samples are included. BH-corrected Wilcoxon-test p-values are denoted. (C) Box plots showing the accessibility of the hormone-like (E) and metabolism (F) communities CpGs for different TCGA-PANCAN cancer types obtained by ATAC-seq. A higher value represents less compact chromatin and vice versa. (PNG) [file pcbi.1012565.s010.png]

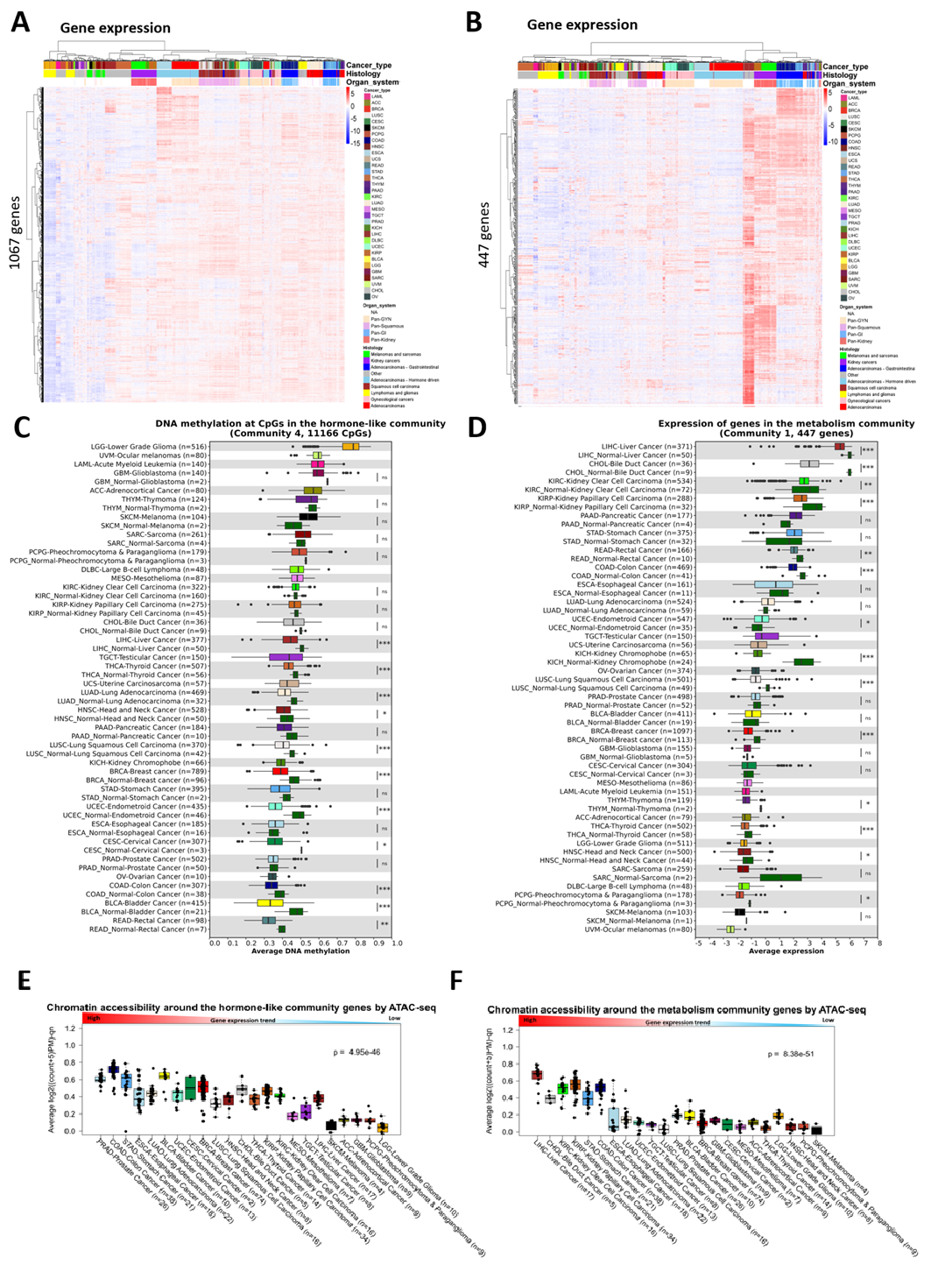

Supplement: S10 Fig — Hierarchical clustering of the expression levels of the genes in the hormone-like (A) and metabolism (B) communities in the TCGA-PANCAN dataset (n = 9875). Rows represent genes and column tumor samples. Red points indicate high expression and blue points indicate low expression. Histopathological features including organ system, cancer type and histology are included. Box plots showing the average expression levels of the hormone-like (C) and metabolism (D) community genes by cancer type. Tumor samples with normal tissue are included. BH-corrected Wilcoxon-test p-values are denoted. Chromatin accessibility around the hormone-like (E) and metabolism (F) community genes in the TCGA-PANCAN tumor samples. (PNG) [file pcbi.1012565.s011.png]

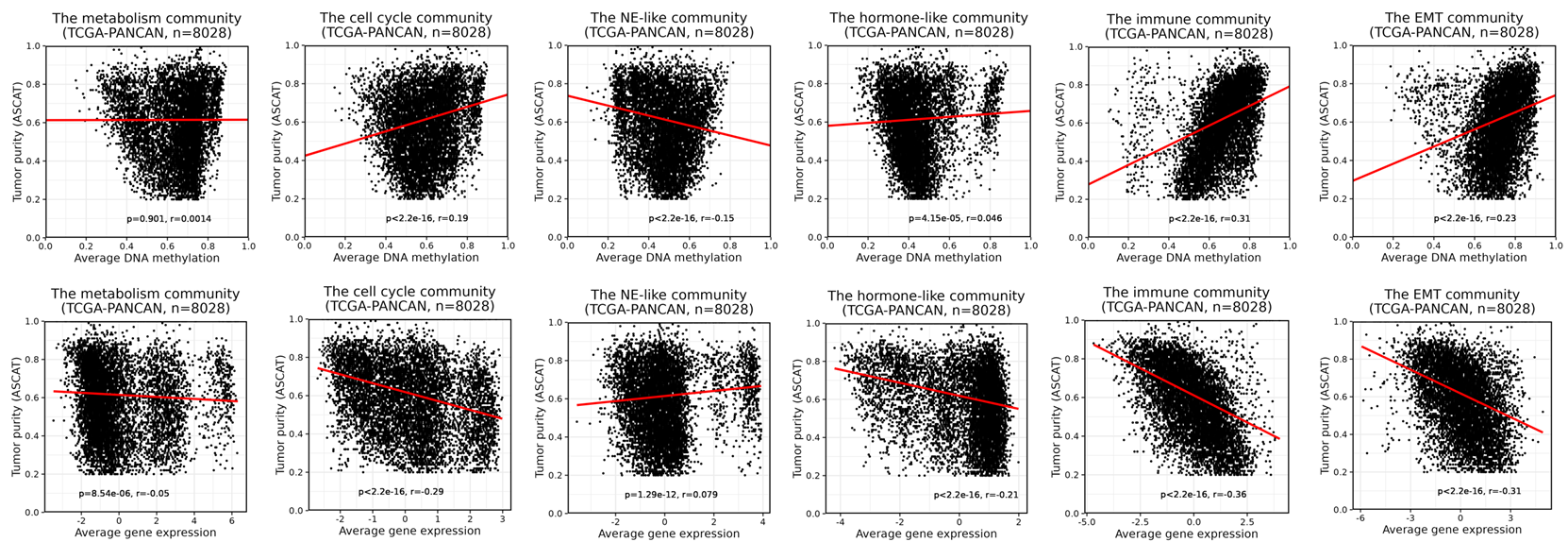

Supplement: S11 Fig — Scatter plots showing the correlation between DNA methylation at the emQTL-CpGs and expression of emQTL genes versus the ASCAT tumor purity estimates in the TCGA-PANCAN dataset. P-values and Pearson correlation coefficients are denoted. (PNG) [file pcbi.1012565.s012.png]

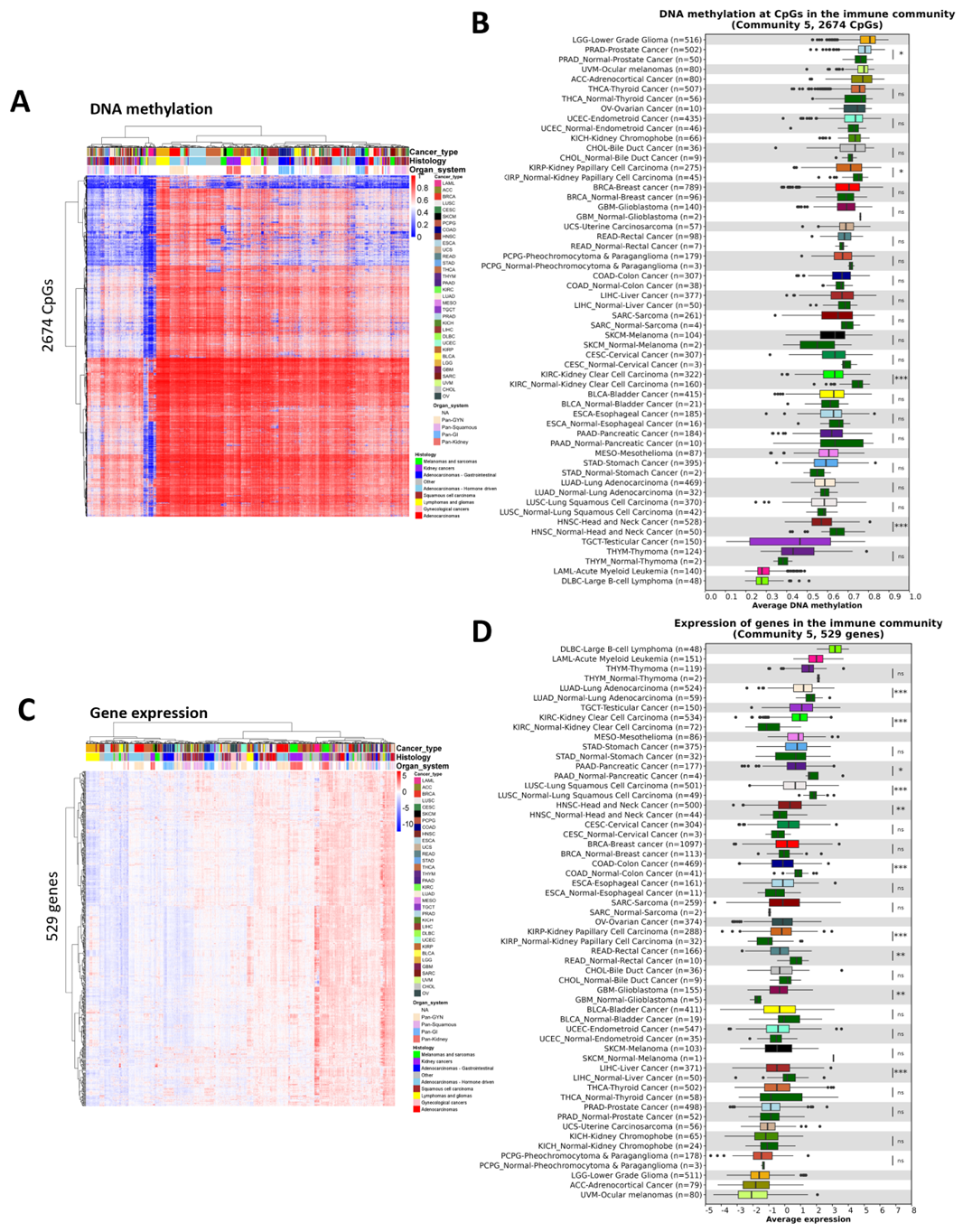

Supplement: S12 Fig — (A) Unsupervised hierarchical clustering of DNA methylation levels at the CpGs in the immune community in TCGA (n = 8229). The red points indicate methylated CpGs and the blue points indicate unmethylated. Rows represent CpGs and columns represent tumor samples. (B) Box plot showing the average DNA methylation levels at the immune community CpGs by TCGA cancer type. DNA methylation levels from available normal samples are included. BH-corrected Wilcoxon-test p-values are denoted. (C) Unsupervised hierarchical clustering of gene expression levels of the immune community genes in each TCGA-PANCAN dataset (n = 9875). Rows represent genes and columns represent tumor samples. Red points indicate high expression and blue points low expression. Histopathological features including organ system, cancer type and histology are included. (D) Box plot showing the average expression levels of the immune community genes by cancer types. Tumor samples with normal tissue are included. BH-corrected Wilcoxon-test p-values are denoted. (PNG) [file pcbi.1012565.s013.png]

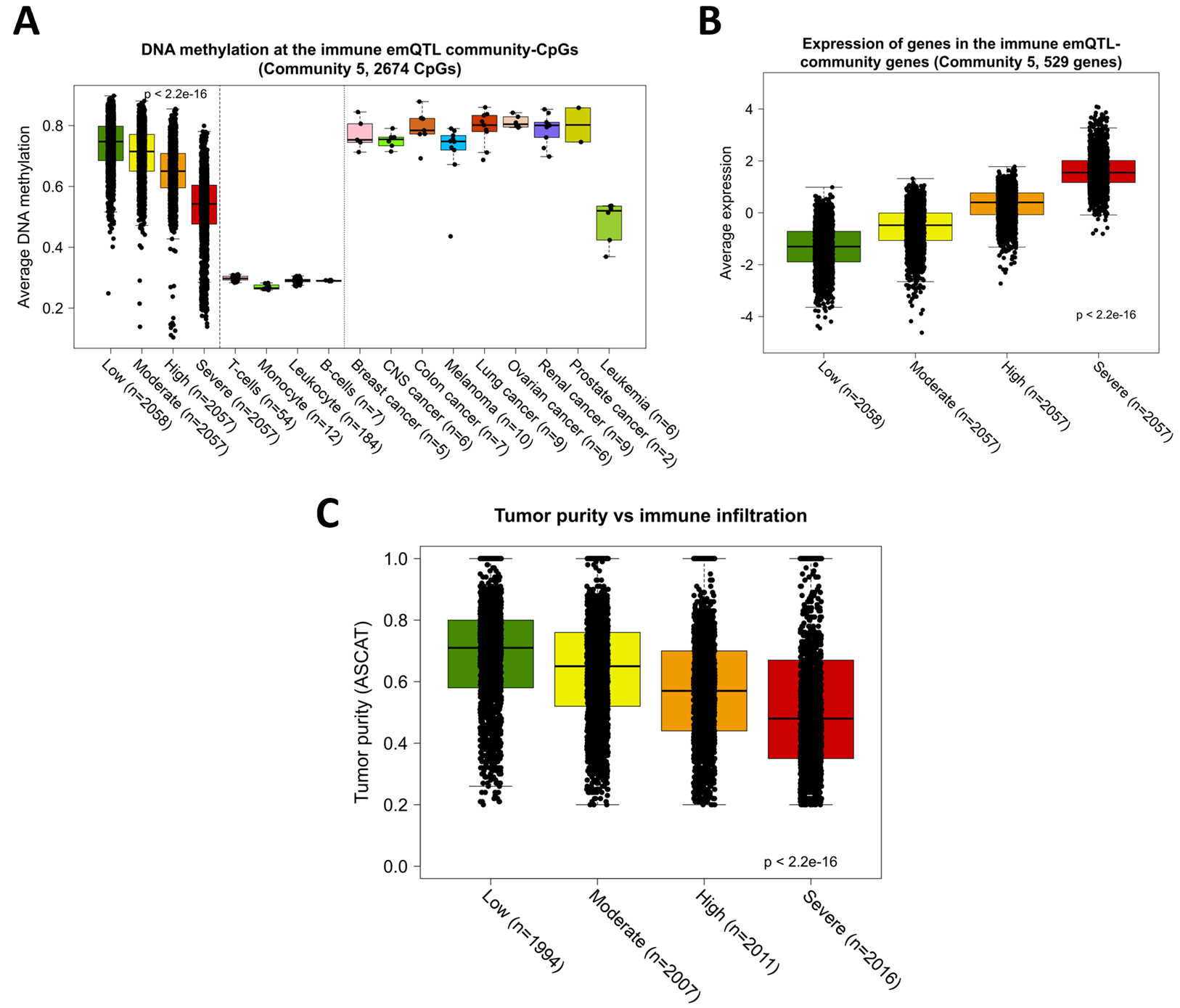

Supplement: S13 Fig — (A) Box plot showing the link between DNA methylation at the immune community CpGs and immune cell infiltration. The level of immune infiltration in tumor samples from TCGA was determined using the xCell deconvolution tool [36]. Tumor samples were divided into quartile groups based on the severity of immune infiltration; low, moderate, high, and severe. DNA methylation levels at the immune community CpGs in isolated immune cells (T-cells, monocytes, B-cells, and Leukocytes) and cancer cell lines from the NCI-60 cancer cell lines are included. (B) Expression of the immune community genes in relation to the xCell-derived immune score. (C) Box plot showing the association between the xCell-derived immune score and tumor purity estimates obtained by ASCAT. Kruskal-Wallis test p-values determined by comparing the quartile groups are denoted in each box plot (A-C). (PNG) [file pcbi.1012565.s014.png]

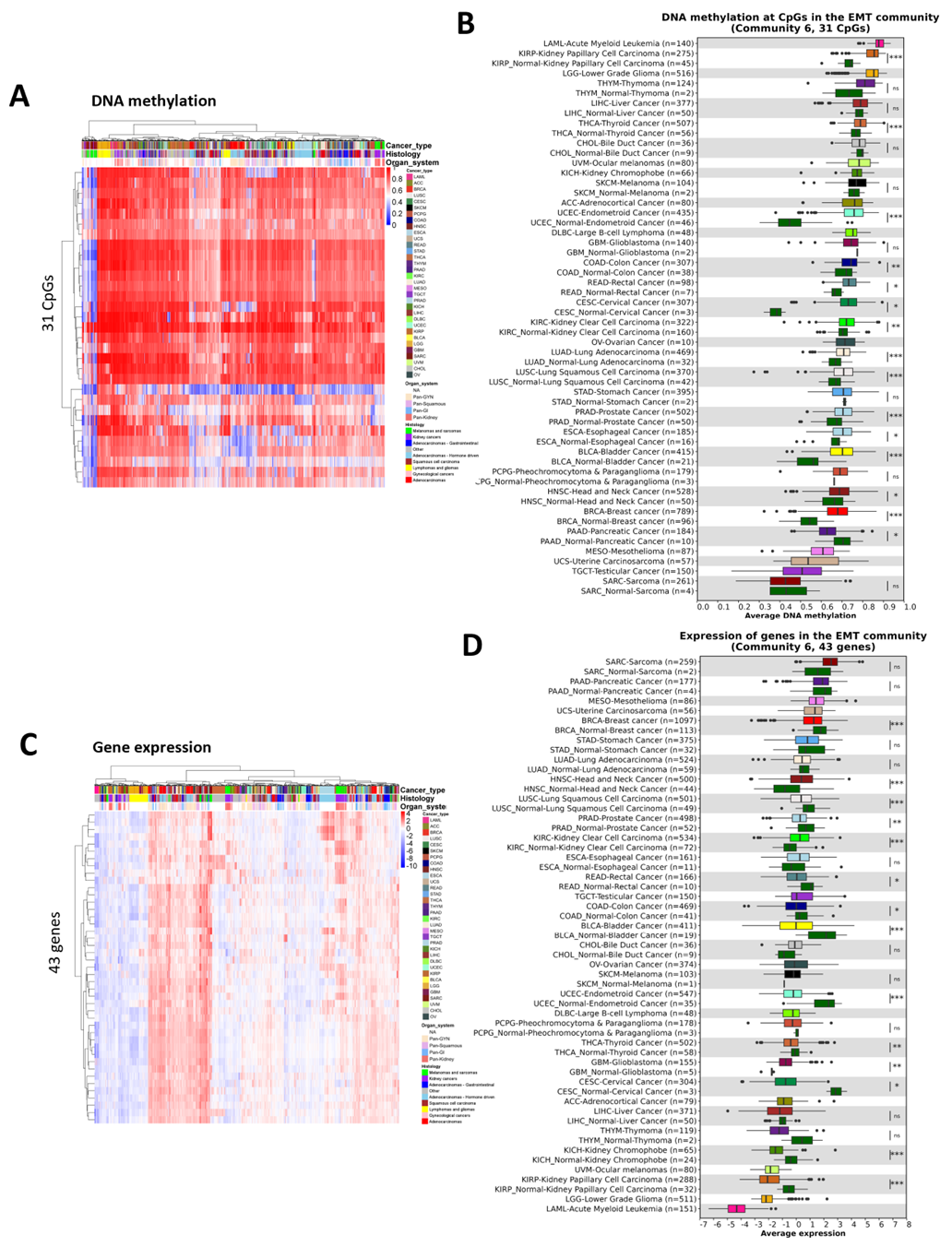

Supplement: S14 Fig — (A) Unsupervised hierarchical clustering of DNA methylation levels at the EMT community CpGs in TCGA-PANCAN (n = 8229). Red points indicate methylated CpGs and blue points indicate unmethylated. Rows represent CpGs and columns represent tumor samples. (B) Box plot showing the average DNA methylation levels at the EMT community CpGs by cancer type. DNA methylation levels from available normal samples are included. BH-corrected Wilcoxon-test p-values are denoted. (C) Unsupervised hierarchical clustering of gene expression levels of the EMT community genes in each TCGA-PANCAN dataset (n = 9875). Rows represent genes and column tumor samples. Red points indicate high expression and blue points indicate low expression. Histopathological features including organ system, cancer type and histology are included. (D) Box plot showing the average expression levels of the EMT community genes by cancer types. Tumor samples with normal tissue are included. BH-corrected Wilcoxon-test p-values are denoted. (PNG) [file pcbi.1012565.s015.png]

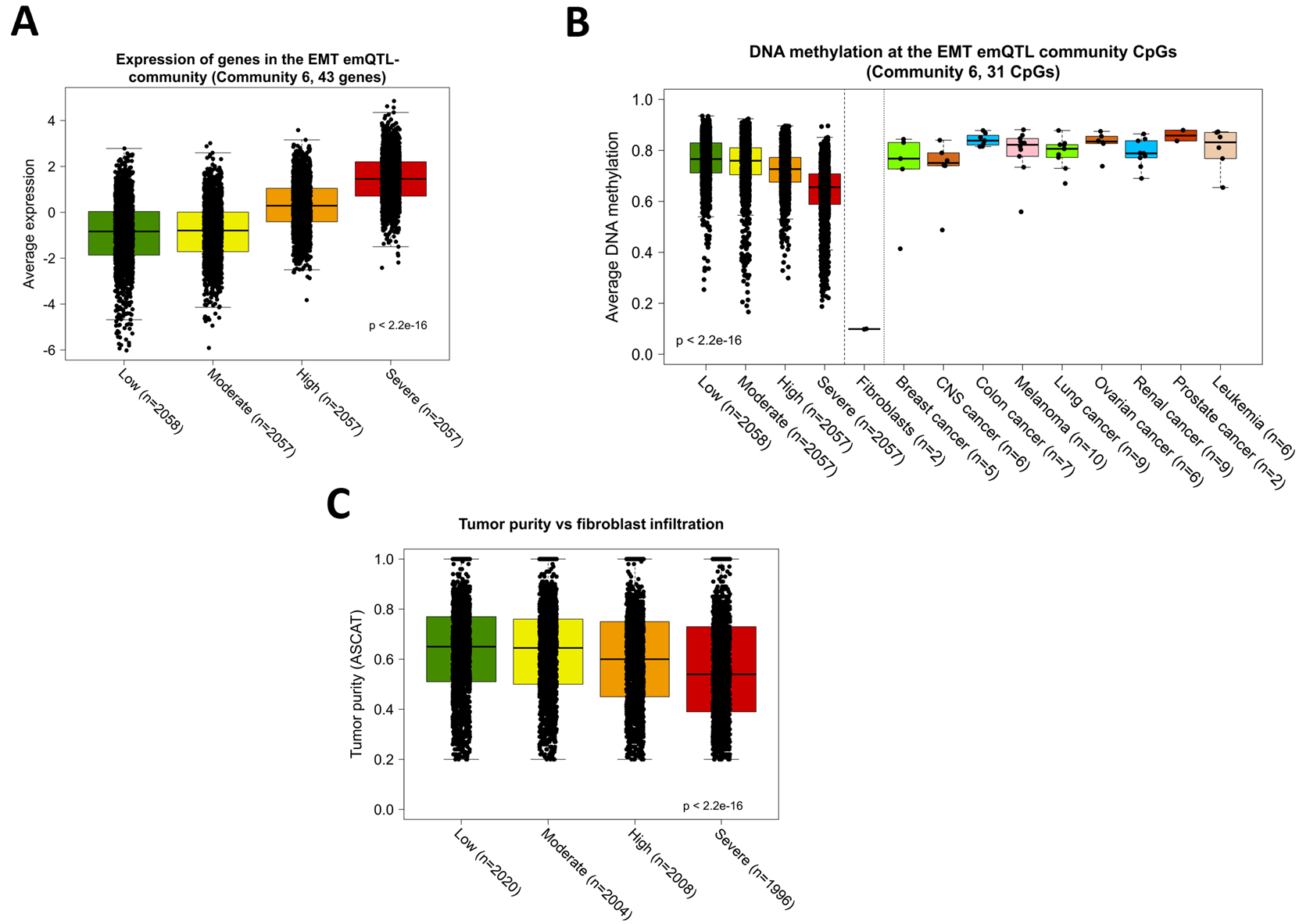

Supplement: S15 Fig — (A) Box plot showing the association between expression of the EMT community genes in relation to fibroblast infiltration. Fibroblast infiltration level in the tumor samples in TCGA was determined by the xCell deconvolution tool. Tumor samples were divided into quartile groups based on the severity of fibroblast infiltration; low, moderate, high, and severe. (B) The association between fibroblast infiltration and DNA methylation at the EMT community CpGs. DNA methylation levels obtained from human mammary fibroblasts and the NCI-60 cancer cell lines are also included. (C) shows the association between fibroblast infiltration and tumor purity estimates from ASCAT. Kruskal-Wallis test p-values comparing the quartile groups are denoted in each plot. (PNG) [file pcbi.1012565.s016.png]

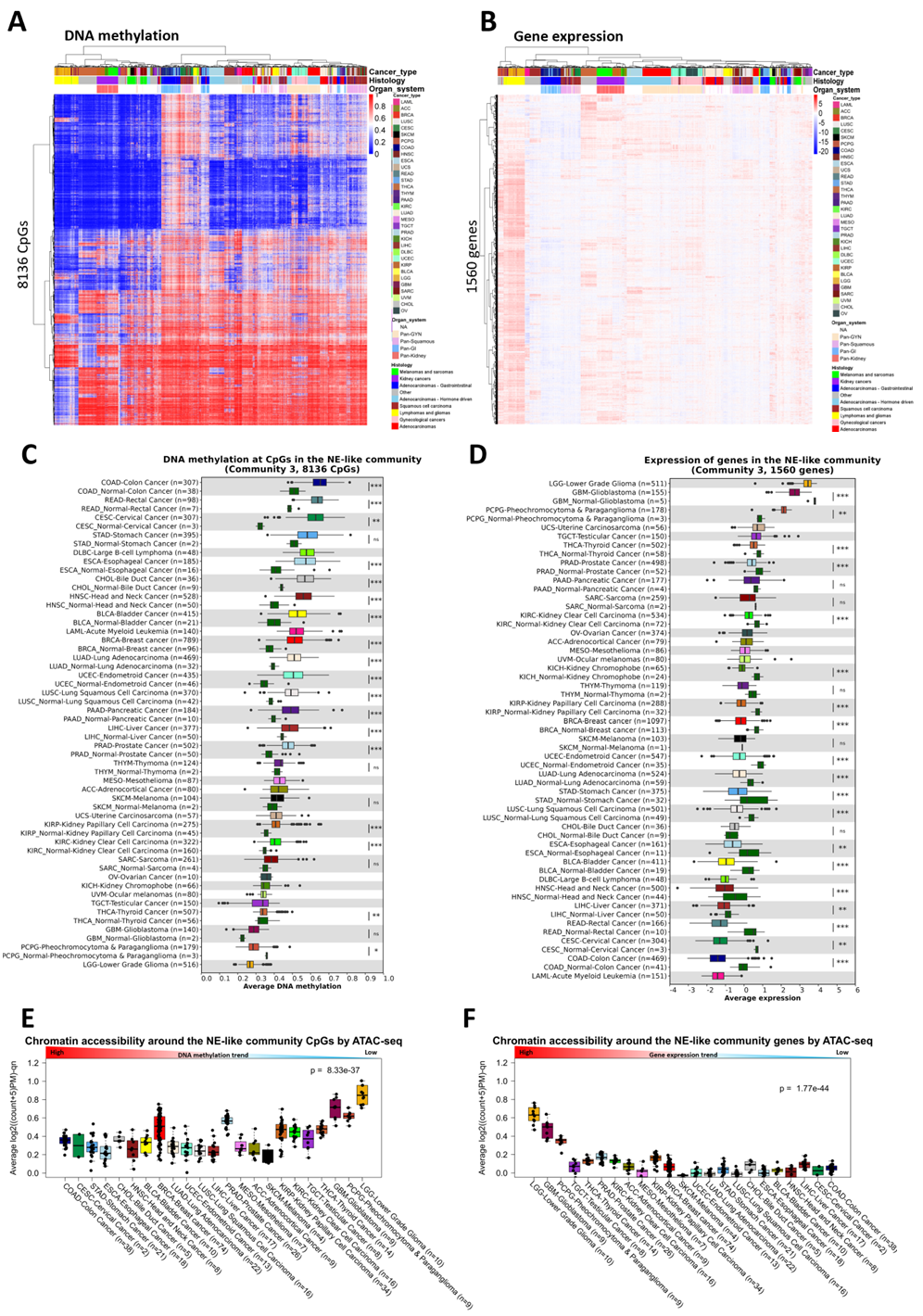

Supplement: S16 Fig — (A) Hierarchical clustering of DNA methylation levels at the NE-like community CpGs in the TCGA-PANCAN dataset (n = 8229). Rows represent CpGs and columns represent tumor samples. Methylated and unmethylated CpGs are shown as red and blue dots respectively. Cancer type, organ system and histology of the cancer type are indicated. (B) Heatmap showing the expression levels of the NE-community genes in the TCGA-PANCAN dataset (n = 9875). Rows and columns represent genes and samples respectively and were ordered by unsupervised hierarchical clustering. Blue points indicate low expression while red points indicate high. The samples are annotated by cancer type, organ system and histology. (C) Box plot showing the average DNA methylation levels at the NE-like community CpGs by cancer type. DNA methylation levels from normal samples are included for those cancer types with data available. BH-corrected Wilcoxon-test p-values are denoted. (D) Box plot showing the average expression of genes in the NE-like community by cancer type. BH-corrected Wilcoxon test p-values are denoted by comparing expression levels of genes between tumor and normal samples. (E) Boxplot showing the chromatin accessibility around the CpGs in the NE-like community as determined by ATAC-seq of the tumor samples from TCGA-PANCAN. A high value represents more open chromatin while a low value represents more compact chromatin. The chromatin accessibility around the NE-like community genes is visualized in (F) for the TCGA-PANCAN dataset. (PNG) [file pcbi.1012565.s017.png]

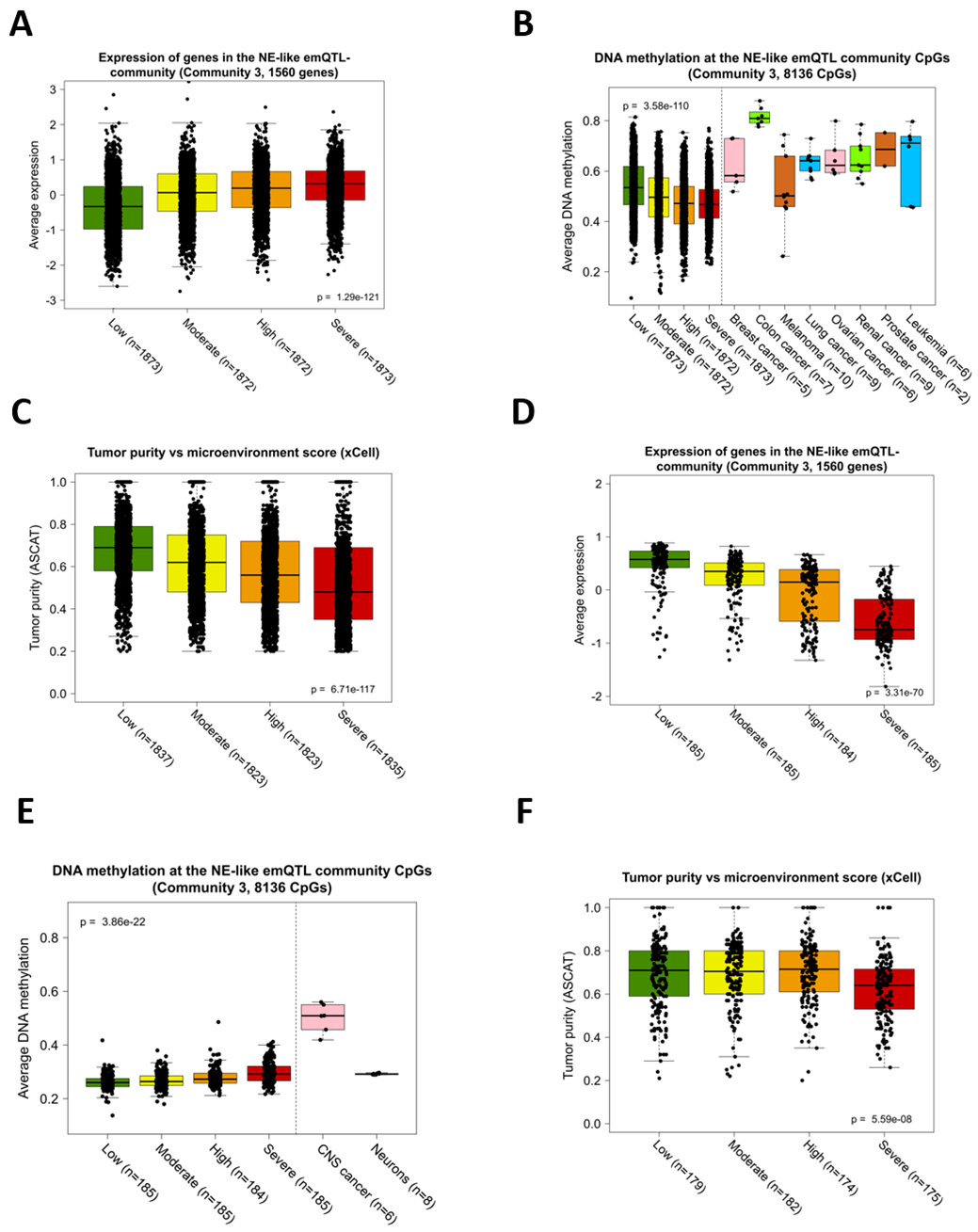

Supplement: S17 Fig — Boxplots showing the expression of the NE-like community genes in the non-neuron (NN) related cancer types (A) and the neuron-related cancer types (LGG, PCPG, GBM; D) in TCGA. The tumors were divided into four quartile groups (Low, moderate, high, and severe) based on the severity of infiltration using the microenvironment score obtained from xCell. The DNA methylation levels at the NE-like community CpGs in NN and non-NN tumors are shown in (B) and (E) respectively. (C) and (F) shows the associations between the microenvironment score and the ASCAT tumor purity score in the non-NN and NN tumors respectively. Kruskal-Wallis test p-values are denoted in each box plot. (PNG) [file pcbi.1012565.s018.png]

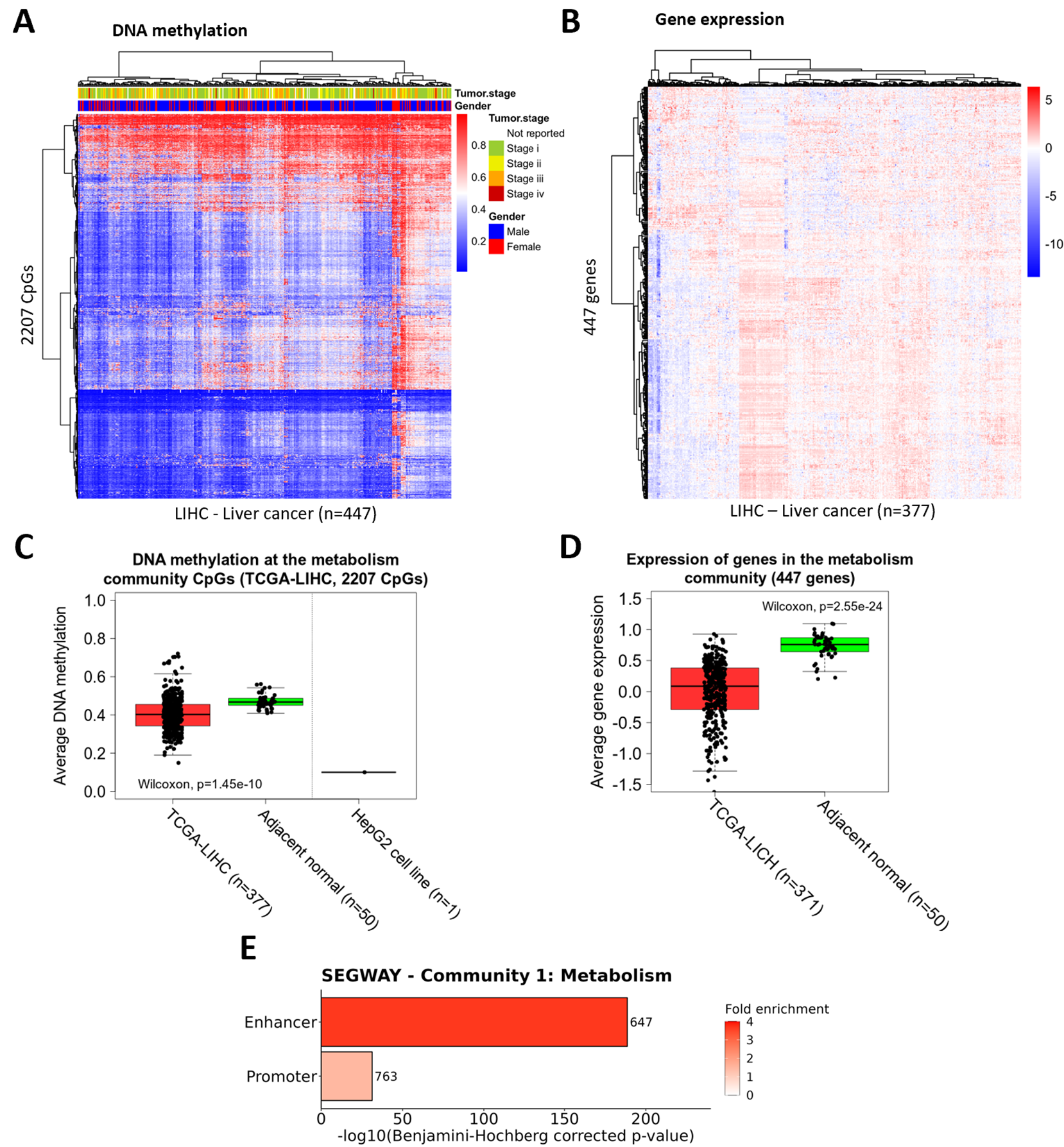

Supplement: S18 Fig — (A) Heatmap showing the level of DNA at the metabolism community CpGs in the TCGA-LIHC dataset (n = 447). Red point indicates methylated CpGs and blue points indicate unmethylated CpGs. Rows represent CpGs and columns represent tumor samples. (B) Unsupervised hierarchical clustering of expression levels of genes in the metabolism community in liver cancer (n = 377). Rows represent genes and columns represent tumor samples. (C) DNA methylation at the CpGs in the metabolism community in liver cancer (TCGA-LIHC). Box plot showing the expression of genes in the metabolism community in liver cancer (TCGA-LIHC). Wilcoxon-rank sum test p-values between tumor and normal samples are denoted in C and D. (E) Enrichment of the metabolism community CpGs in LIHC-defined regulatory regions obtained by Segway. The length of the bars represents -log10-transformed p-values obtained by hypergeometric testing using all the 450k probes as background. Only significant enrichments (BH-corrected p-value<0.05) are shown. (PNG) [file pcbi.1012565.s019.png]

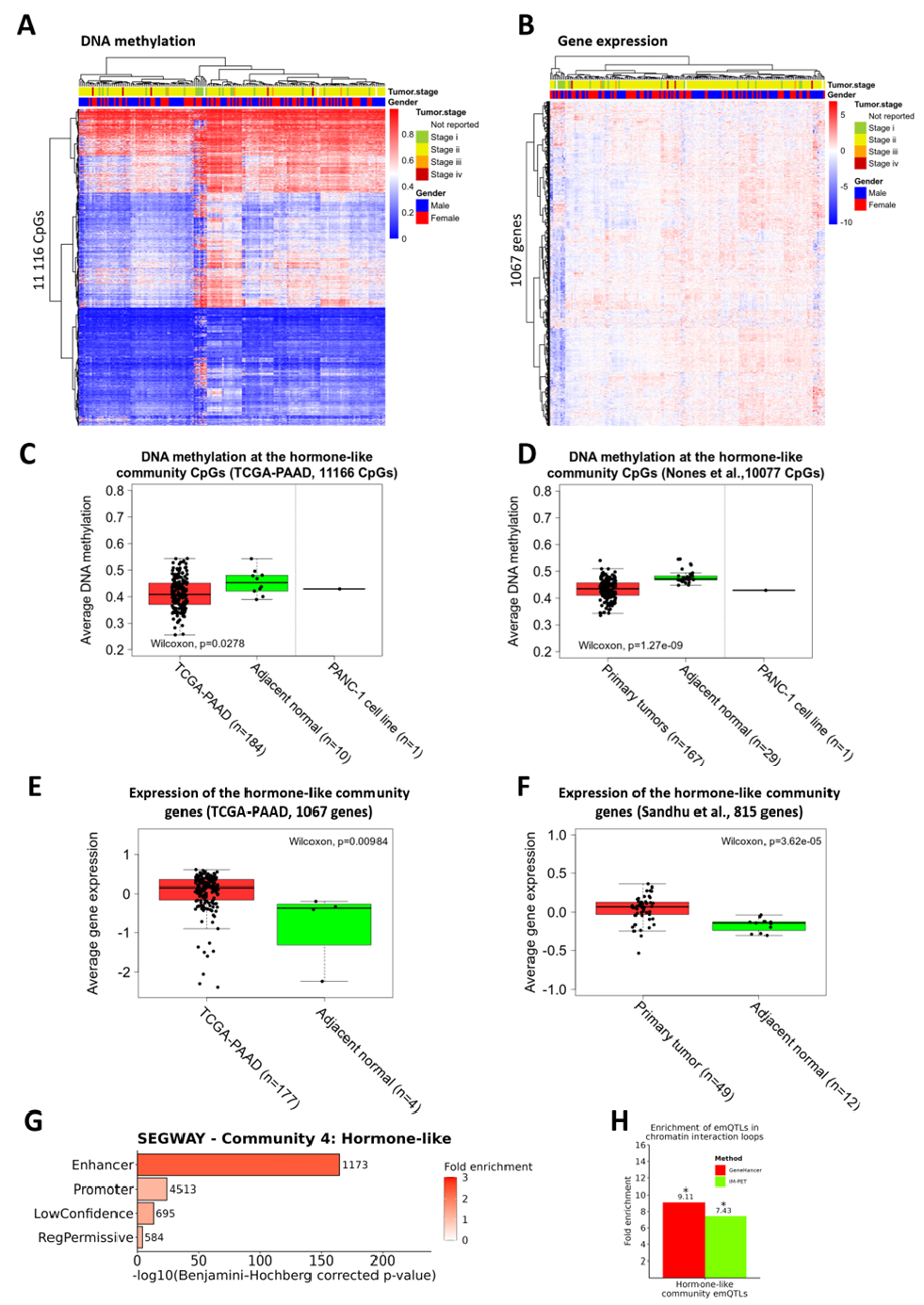

Supplement: S19 Fig — (A) Unsupervised hierarchical clustering of the DNA methylation levels at the hormone-like community CpGs in pancreatic cancer (TCGA-PAAD). (B) Hierarchical clustering of the expression levels of the hormone-like community genes in pancreatic cancer (TCGA-PAAD). (E-F) Box plot showing the DNA methylation levels at the CpGs in the cell cycle community in TCGA (C) and the independent pancreatic cancer dataset obtained from Nones et al. [23] (D). DNA methylation levels for the PANC1 cell line are also included. Expression of the hormone-like community genes in pancreatic tumors from TCGA (E) and an independent cohort obtained from Sandhu et al. [24] (F). Wilcoxon-rank sum test p-values are denoted in each box plot. (G) Bar plot showing the enrichment of the hormone-like community CpGs in different regulatory regions of the genome according to SEGWAY from PANC1. (H) Enrichment of emQTL in the hormone-like community in IM-PET chromatin loops from the PANC1 pancreatic cancer cell line. The enrichment was determined using hypergeometric tests of significance using all possible in cis CpG-gene pairs as background. Significant enrichments after BH correction are marked with an asterisk. (PNG) [file pcbi.1012565.s020.png]
